# Sub-nanometre control of the coherent interaction between a single molecule and a plasmonic nanocavity

**DOI:** 10.1038/ncomms15225

**Published:** 2017-05-19

**Authors:** Yao Zhang, Qiu-Shi Meng, Li Zhang, Yang Luo, Yun-Jie Yu, Ben Yang, Yang Zhang, Ruben Esteban, Javier Aizpurua, Yi Luo, Jin-Long Yang, Zhen-Chao Dong, J G Hou

**Affiliations:** 1 Hefei National Laboratory for Physical Sciences at the Microscale and Synergetic Innovation Center of Quantum Information and Quantum Physics, University of Science and Technology of China, Hefei 230026, China; 2 Materials Physics Center (CSIC-UPV/EHU), Paseo Manuel de Lardizabal 5, Donostia-San Sebastián 20018, Spain; 3 Donostia International Physics Center (DIPC), Paseo Manuel de Lardizabal 4, Donostia-San Sebastián 20018, Spain; 4 IKERBASQUE, Basque Foundation for Science, Maria Diaz de Haro 3, Bilbao 48013, Spain

## Abstract

The coherent interaction between quantum emitters and photonic modes in cavities underlies many of the current strategies aiming at generating and controlling photonic quantum states. A plasmonic nanocavity provides a powerful solution for reducing the effective mode volumes down to nanometre scale, but spatial control at the atomic scale of the coupling with a single molecular emitter is challenging. Here we demonstrate sub-nanometre spatial control over the coherent coupling between a single molecule and a plasmonic nanocavity in close proximity by monitoring the evolution of Fano lineshapes and photonic Lamb shifts in tunnelling electron-induced luminescence spectra. The evolution of the Fano dips allows the determination of the effective interaction distance of ∼1 nm, coupling strengths reaching ∼15 meV and a giant self-interaction induced photonic Lamb shift of up to ∼3 meV. These results open new pathways to control quantum interference and field–matter interaction at the nanoscale.

## Abstract (web summary)

Assessing the coupling between a plasmonic nanocavity and a single quantum emitter is challenging due to the lack of spatial control at the atomic scale. Here Zhang *et al*. achieve control with sub-nanometre precision and demonstrate the Fano resonance and Lamb shift at the single-molecule level.

The coherent interaction between a quantum emitter and a resonant photonic cavity is of paramount importance in the development of photon-based quantum information technologies^1^,^2^ as well as in molecular sensing and spectroscopy^3^,^4^,^5^,^6^,^7^,^8^,^9^,^10^. The properties of the cavity determine the transition rate and the energy shift of the emission^11^. The plasmonic nanocavity is particularly attractive in this regard because of its capability to squeeze the optical fields down to the nanoscale, with much enhanced local density that promotes the coupling of the cavity with a single emitter, in spite of ohmic losses^12^,^13^,^14^. When a quantum emitter is within a resonant plasmonic nanocavity, quantum interference may occur due to the coherent interaction between the discrete state of the emitter and the continuum-like state of the cavity, resulting in distinctive spectral features called Fano resonances^15^,^16^,^17^,^18^. This coherent interaction could also affect the energy level of the quantum emitter itself and result in a shift of optical transition energy, known as the photonic Lamb shift^19^,^20^,^21^. However, the attempt to actively tune Fano resonances and Lamb shifts of a single quantum emitter in a nanocavity by optical spectroscopies has been elusive up to date.

Through the use of a single molecule as a quantum emitter and precise spatial control of its position with respect to the plasmonic nanocavity formed by the metal tip and substrate in a scanning tunnelling microscope (STM), one can build up an ideal platform not only to confine and enhance the local electric field at the nanoscale but also to tailor the coherent interaction between the molecule and cavity at the atomic scale^22^,^23^. Furthermore, the highly localized tunnelling electrons can also act as a source of excitation for light, a technique generally called STM-induced luminescence (STML) and capable of achieving spectral characterization with spatial resolution down to sub-nanometre scale^24^,^25^,^26^,^27^.

In this work, we use this technique to reveal the Fano resonance resulting from the coherent coupling between an electronically decoupled single zinc-phthalocyanine (ZnPc) molecule and a plasmonic nanocavity in close proximity and demonstrate how the spatial dependence of the single-molecule Fano resonance and Lamb shift can be used to quantitatively retrieve the intrinsic properties of the nanocavity plasmon and field–matter interaction.

## Results

### Single-molecule Fano resonance

**Figure 1:**
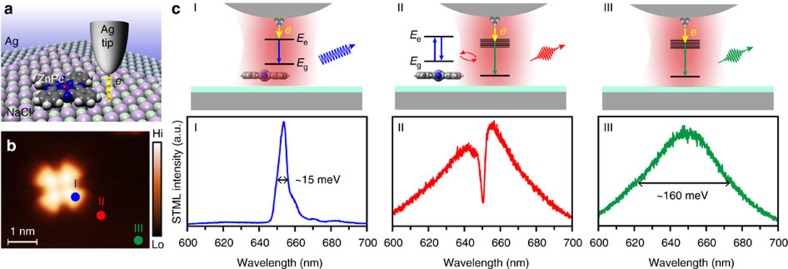
Realization of single-molecule Fano resonance. (**a**) Schematic experimental setup to achieve single-molecule Fano resonance. (**b**) STM topograph of a single ZnPc molecule on the NaCl island acquired at −1.7 V and 2 pA. (**c**) Three different junction structures (top schematics) and corresponding STML spectra (bottom, −2.5 V, 200 pA, 20 s). Situation I: on top of the molecule (blue dot in (**b**)); Situation II: in close proximity to the molecule (red dot in (**b**)); Situation III: far away from the molecule (green dot in (**b**)). A typical Fano lineshape is observed in situation II due to the interaction between the nanocavity plasmon and the two-level emitter in close proximity. The red halos in the schematics represent localized plasmonic field. See text for details.

Figure 1a shows the experimental setup to achieve single-molecule Fano resonance by tunnelling electron excitation for a single ZnPc molecule in close proximity to a plasmonic nanocavity defined by a silver (Ag) tip and Ag(100) substrate, under low-temperature (∼8 K) and ultrahigh-vacuum (1 × 10^−10^ Torr) conditions (see Methods section for more experimental details). The ZnPc molecule is electronically well decoupled from the underlying metal substrate by three NaCl monolayers, which ensures the single molecule to behave as a well-defined two-level quantum emitter through suppressing the rapid non-radiative decay channels and resultant fluorescence quenching^24^. The decoupled ZnPc molecule shows a four-lobe pattern in STM imaging (Fig. 1b) that agrees with the molecular structure^24^. Depending on the position of the tip with respect to the molecule, the photon emission spectra excited by the tunnelling electrons are dramatically different owing to different interaction and excitation regimes involved. When the tip is positioned above the molecule (blue dot in Fig. 1b and situation I in Fig. 1c), the molecule is directly excited by the injected electrons, thus giving rise to sharp molecule-specific fluorescence of ZnPc at ∼651 nm (∼1.905 eV)^24^. When the tip is positioned at a certain distance away from the molecule (for example, >3 nm, schematically shown as the green dot in Fig. 1b and situation III in Fig. 1c), a broad plasmonic emission band around 650 nm (∼1.908 eV) is observed, which is known to arise from the radiative decay of the nanocavity plasmon excited by inelastic tunnelling electrons^25^,^26^,^27^,^28^,^29^. Remarkably, when the tip is positioned in close proximity to the molecule (red dot in Fig. 1b and situation II in Fig. 1c), the emission spectrum shows a clear dip superimposed over the broad plasmonic spectrum, with the dip position closely associated with the emission peak energy of the ZnPc molecule. Such spectral feature is characteristic of the Fano lineshape, revealing the coherent interaction between the sharp discrete molecular transition of frequency (*ω*_m_) and the broad-continuum-like plasmonic resonance of frequency (*ω*_p_)^6^,^15^,^16^,^30^,^31^,^32^. It is worthy to point out that, in situation II, the tunnelling electrons excite only the nanocavity plasmon, rather than the molecule directly because of the highly localized nature of tunnelling electrons; the molecule can then be excited indirectly by coupling with the nanocavity plasmon in close proximity. Such coupling enables the quantum interference between two paths of emission through either the molecular transition or the plasmon resonance, leading to the Fano lineshape observed. We would like to note that electrically driven Fano resonance can also be observed for a gold nanoparticle in the gap between two electrodes^17^. In our work, the feature of the Fano lineshape can be modulated spatially by modifying the coupling strength between the molecule and nanocavity plasmon, and spectrally by the energy detuning between the molecular transition and plasmon resonance, Δ=*ω*_p_−*ω*_m_, as we will explain in detail below.

### Distance-dependent coupling strength

**Figure 2:**
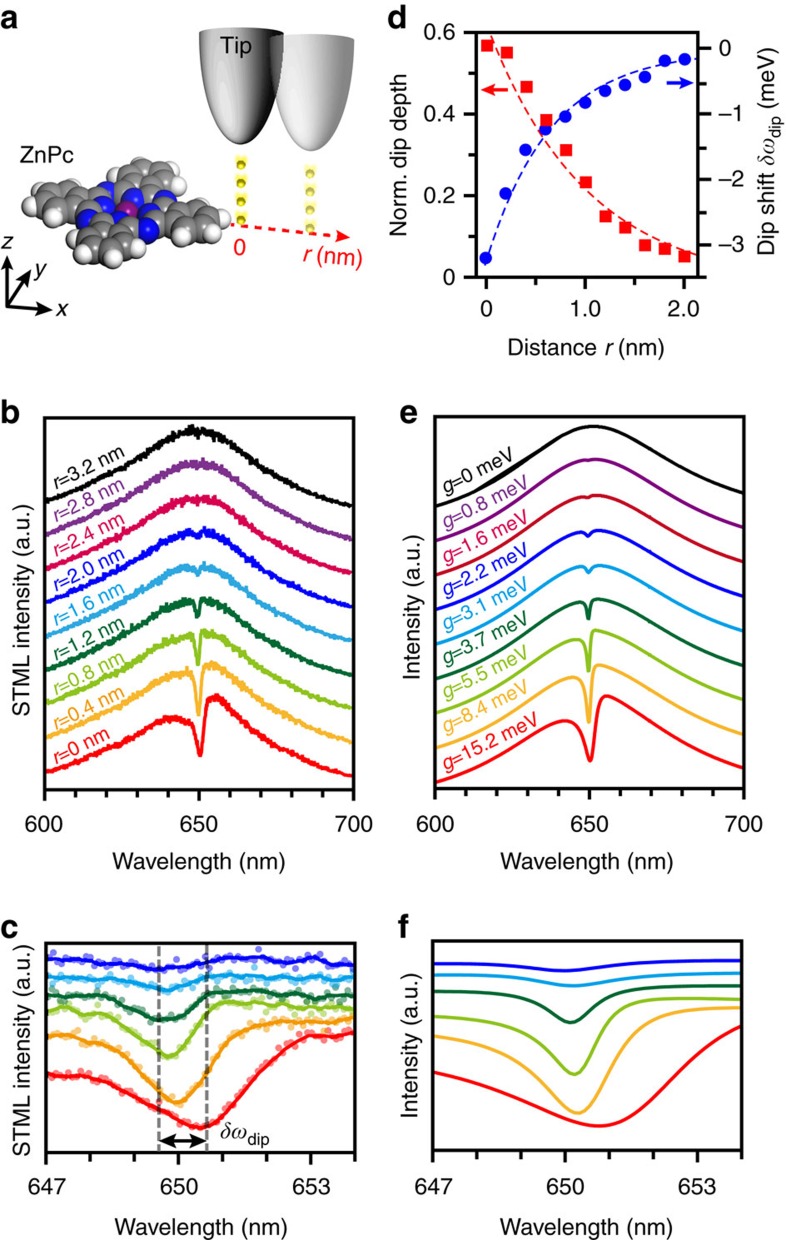
Distance dependence of single-molecule Fano resonance. (**a**) Schematics showing the tip trajectory of STML measurements with the definition of the position for *r*=0 at the edge of the ZnPc molecule. (**b**) A series of experimental STML spectra along the line trace in (**a**) (−2.5 V, 200 pA, 20 s) with a lateral separation distance from *r*=0 to *r*=3.2 nm. (**c**) Enlarged spectra of (**b**) highlighting the dip shift (*δω*_dip_) for *r*=0 to *r*=2 nm. (**d**) Variation of the normalized dip depth (*D*_norm_) and dip shift (*δω*_dip_) with lateral separation distances. (**e**) Simulated spectra showing the coupling strength (*g*). (**f**) Enlarged spectra of (**e**) highlighting the dip shift.

The coupling strength of this coherent interaction can be controlled on demand, thanks to the precise spatial control of the relative distance between the molecule and the plasmonic cavity. The emission spectral features can be monitored while the tip is moved away from the edge of the molecule along a path schematically shown in Fig. 2a, where the point of maximum proximity to the molecule is defined as *r*=0 through the abrupt change of spectral features from a clear molecular fluorescence (spectrum I in Fig. 1c) to the Fano lineshape (spectrum II in Fig. 1c) (Supplementary Note 1).

Figure 2b shows an example of evident spectral evolution when the tip is moved along the lobe direction of the molecule. As the tip gets closer to the molecule *(r*→0), the Fano dip is deepened and widened (from black to red curves), which indicates an increase of the coupling strength between the nanocavity plasmon and the ZnPc molecule. A detailed inspection of the Fano spectra not only shows the increase of the depth of the Fano dip but also reveals a systematic shift in the dip position (Fig. 2c) especially when the tip is in the proximity of the molecule within 1 nm. A quantitative analysis of both the normalized dip depth (*D*_norm_) and the spectral shift (*δω*_dip_) of the Fano dip are shown in Fig. 2d as a function of tip (plasmonic nanocavity) positions (*r*). The normalized dip depth is found to decay exponentially with increased separation. An exponential fit, *D*_norm_∝***e***^−*r/l*^, determines a decay length *l* as small as ∼0.9(1) nm, which suggests that the effective interaction distance of the plasmonic field is highly confined within ∼1 nm. The spectral shift of the Fano dip also exhibits an approximately exponential dependence on the separation between the molecule and the plasmonic nanocavity, with a maximum shift of ∼3 meV.

It should be noted that the situation in Fig. 2b corresponds to the zero-detuning condition (Δ=0). The shift of the Fano dip for zero detuning is surprising if both the molecule and plasmon are regarded as harmonic oscillators within a dipole approximation. In such case, the self-interaction of the molecule via the interaction with the plasmon would be described by a pure imaginary Green's function, which would not reproduce the frequency shift experimentally observed (Supplementary Note 2). The Fano dip position would then be determined by the intrinsic properties of the electronic transition of the molecule alone^33^. The observation of dip shifts along with the position-dependent change of coupling strength suggests the involvement of additional effects. Indeed, in a highly confined plasmonic nanocavity, the self-interaction of the molecule with the strongly enhanced plasmonic field in the nanocavity goes beyond the simple dipole model, resulting in a considerably large shift of the energy level of the molecule itself identified as the so-called photonic Lamb shift^34^,^35^.

The evolution of the experimentally observed spectra in Fig. 2b,c can be well reproduced by introducing the self-interaction term into the harmonic oscillator model. This framework provides an expression for the radiative power, *P*(*ω*), in terms of the square of the effective dipole moment of the plasmon, **p**_p_ (Supplementary Note 3) as:


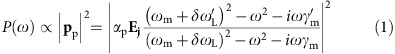


in which *ω* is the (angular) frequency, *α*_p_ represents the polarizability of the plasmon dipole, *ω*_m_ the transition frequency of the molecule, *δω*_L_ the Lamb shift and 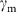 the total decay rate of the molecule; 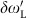 and 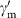 are, respectively, an energy shift and decay rate associated with the influence of the complex plasmonic fields. Equation (1) can be rewritten in terms of a Fano-line profile^16^, 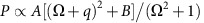, where 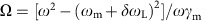 is a dimensionless frequency and 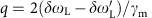 is the Fano parameter (see Supplementary Note 3 for details).

By applying equation (1) to describe the Fano profiles, we can nicely reproduce the experimental spectra regarding both the variation of the dip depth and the shift of the dip position (Fig. 2e,f), allowing to quantitatively estimate both the coupling strength and the Lamb shift revealed in each spectrum, respectively. The effective coupling strength *g* is related to the normalized Fano dip depth *D*_norm_ as 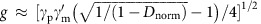, where *γ*_p_ is the decay rate of the plasmon (see Supplementary Note 4 for more details). The coupling strength can reach a value as large as ∼15 meV, whereas the maximum value of the Lamb shift reaches ∼3 meV when the tip is placed at the closest proximity of the ZnPc molecule (*r*=0). Since the ZnPc molecule has a transition dipole moment of 10.6 Debye^24^,^36^ and a resultant spontaneous decay rate of 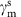
∼0.128 ns^−1^ (that is, ∼0.0841 μeV), the obtained coupling strength of ∼15 meV corresponds to an effective Purcell factor up to about 6.9 × 10^4^ estimated via 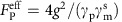
37. Note that the Lamb shift of ∼3 meV is much larger than the vacuum Lamb shift^19^,^20^,^38^, due to the strongly enhanced local field in the plasmonic nanocavity. We would like to point out that Lamb shifts can also be observed from the spectral shifts of the Fano dip when the tip is moved vertically away from a single molecule (see Supplementary Note 5).

### Fano resonance for different energy detunings

**Figure 3:**
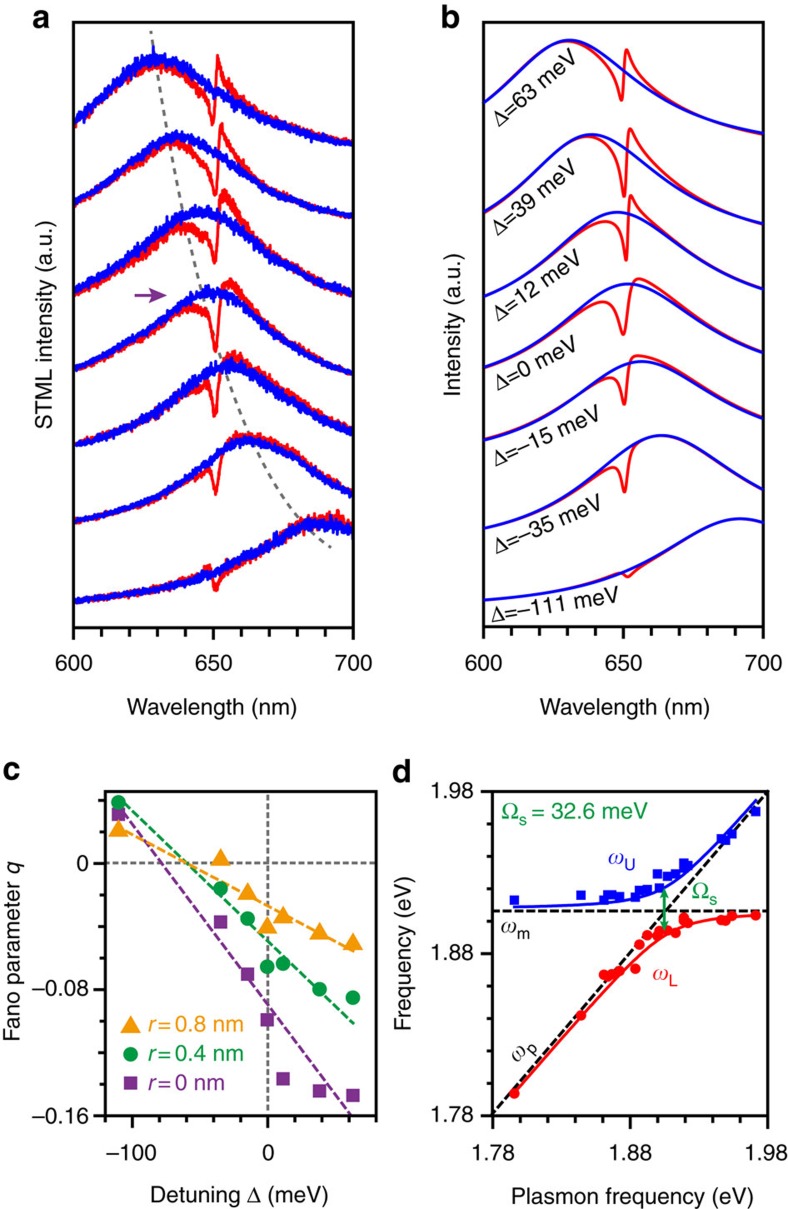
Evolution of the single-molecule Fano spectra with different energy detunings. (**a**) Experimental STML spectra (−2.5 V, 200 pA, 20 s) showing pristine nanocavity plasmon mode (blue lines, measured with the tip far away from the molecule) and the corresponding Fano resonance (red lines) for different detunings. The situation of zero detuning is marked with an arrow. (**b**) Simulated Fano spectra (red lines) for different detunings from −111 to 63 meV. The plasmonic background spectra (blue lines) are introduced by Lorentzian lineshapes with the peak position determined by the different detunings accordingly. (**c**) Variation of Fano parameter *q* as a function of detuning for different tip positions: *r*=0 (square), *r*=0.4 nm (circle), and *r*=0.8 nm (triangle). The purple, green and orange dashed lines are linear fits to the corresponding data. (**d**) Peak-maximum positions of the experimental Fano spectra as a function of the plasmonic frequency. Blue and red dots correspond to the upper- and lower-energy peaks (denoted as *ω*_U_ and *ω*_L_), respectively. Two solid lines are theoretically fitted curves (Supplementary Note 6). The dashed lines represent the positions of the plasmonic frequency *ω*_p_ and the molecular transition frequency *ω*_m_. The energy separation at zero detuning, Ω_s_, is marked in green.

We have so far considered the coherent coupling between the single molecule and the nanocavity plasmon for a situation of zero detuning (Δ=0). A great advantage of the plasmonic nanocavity in the STM is that, by modifying the tip shape and morphology, the resonant frequency of the nanocavity plasmon can be tuned with respect to the frequency of the molecular emission, allowing extra degrees of control of the coherent interaction in the Fano resonance^13^. In the present work, the resonance wavelength of the nanocavity plasmon is tuned from 620 to 690 nm, as shown in the blue spectra of Fig. 3a. The molecular transition for each of these situations, however, is kept at a wavelength of ∼651 nm. The experiments for different detuning conditions were carried out at the same tip position that was selected to be in the closest proximity to the ZnPc molecule (*r*=0 position in Fig. 2a). Figure 3a plots a series of emission spectra in red showing clear Fano lineshape, which is found to strongly depend on the detuning between the plasmonic and molecular emission and exhibits varying asymmetric spectral features. By applying equation (1) to different detuning conditions (Δ ranging from +63 to −111 meV), the evolution of the spectral features is well reproduced, as shown in Fig. 3b, stressing the importance of the molecule's self-interaction in the strong plasmonic field.

The asymmetry of the Fano lineshape can be addressed through the Fano parameter *q* introduced earlier (Supplementary Note 2). This parameter varies from negative to positive values for asymmetric Fano profiles, with a value of zero corresponding to a symmetric dip shape^16^. Within the dipole approximation for the coupled oscillator model, the value of *q* is proportional to the detuning Δ (see Supplementary Note 6 for details). Therefore, at zero detuning (Δ=0), the Fano parameter *q* should also be zero, which would lead to a perfectly symmetric dip^21^,^34^,^35^. However, we find experimentally an evident asymmetric Fano lineshape even for zero detuning, as illustrated in the spectrum marked with an arrow in Fig. 3a. Such an asymmetry reveals that the plasmon–molecule interaction in our system goes beyond a simple dipolar interaction picture.

In order to further explore this asymmetry quantitatively, the variation of the Fano parameter *q* with detuning is plotted in Fig. 3c for three different tip–molecule separations at *r*=0, 0.4 and 0.8 nm, respectively. The dashed lines are a linear fit of the data considering that the value of *q* is proportional to the detuning. In all three cases, the Fano parameter *q* is always non-zero for Δ=0, indicating that the asymmetric Fano lineshape at zero detuning is given by the inherent complexity of the coherent coupling that involves the spatial distribution of the molecular transition dipole and higher-order plasmonic modes. The change in the asymmetry of the Fano lineshape is also revealed by the observed variations of the Fano parameter from positive to negative values as the energy detuning is modified. Such change, together with the coherent nature of the coupling in Fano resonance, reveals that the broadband nanocavity plasmon has to be regarded as a continuum-like state and acts as a coherent source to excite and interfere with the discrete molecular transition.

The evolution of the Fano lineshape for different detunings can also be used to retrieve the coupling strength. Although the interaction between the nanocavity plasmon and the molecule here is in the weak coupling regime, not strong enough to result in a peak splitting with two hybridized states, the two peak-maximum positions in the emission spectra can be used to estimate the magnitude of this coupling strength. As shown in Fig. 3d for the closest proximity situation (*r*=0), an ‘anti-crossing'-like feature can be identified, yielding an energy ‘splitting' (Ω_s_) of ∼32.6 meV. This value is approximately twice the value of the coupling strength (*g*) obtained from the dip depth in Fig. 2e, fulfilling the generic relationship (Ω_s_=2 *g*) commonly found for systems in the strong coupling regime. Note that the ‘splitting' energy of 32.6 meV obtained is of the same order of magnitude as the Rabi splitting (80–95 meV) reported in the strong coupling regime for a single molecule^3^. Such a magnitude of the coupling strength (∼15 meV), together with a giant Lamb shift (∼3 meV), suggests that the interaction achieved here is approaching the strong coupling regime, which facilitates the identification of the Fano profile of even a single molecule.

### Orientation dependence of the photonic Lamb shift

**Figure 4:**
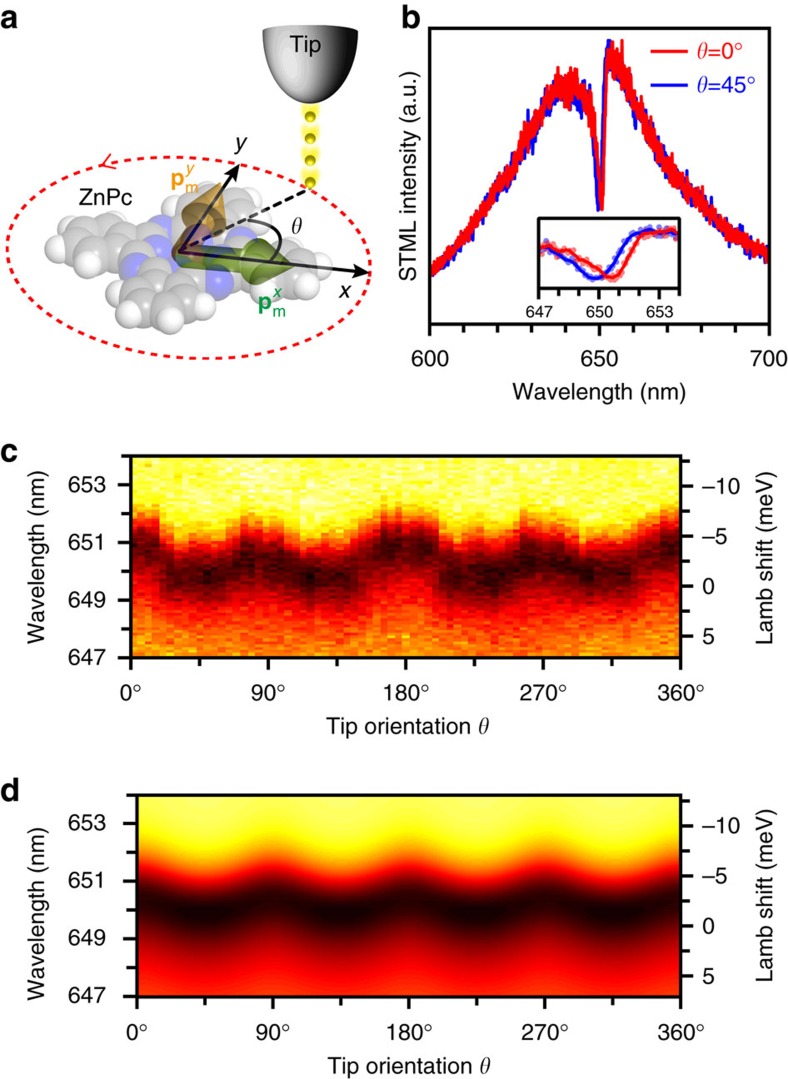
Orientation dependence of the Lamb shift. (**a**) Schematics showing orientation-dependent measurement of Fano spectra. The red dashed line shows the trajectory of the STM tip around the ZnPc molecule, and the green and orange arrows represent two degenerate transition dipole moments 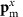 and 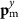 of the molecule, respectively. (**b**) Two typical Fano spectra (−2.5 V, 200 pA, 5 s) for the tip orientation at *θ*=0° (lobe) and *θ*=45° (in between lobes), respectively. (**c**) Colour plot of experimental STML spectral intensity around the Fano dip showing periodic variations of the Lamb shift over *θ*=0−360°. (**d**) Theoretical simulation of the Fano spectra in (**c**), taking into account the periodic variation of the molecule's self-interaction (Lamb shift).

We consider now the vectorial nature of both the molecular transition dipole moment and the local plasmonic field. The coherent coupling between the molecule and the nanocavity plasmon could also be influenced if the STM tip is placed in different directions with respect to the single molecule. Taking into account the D_4h_ symmetry of the ZnPc molecule and the associated two equivalent orthogonal transition dipole moments (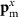 and 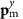) along the lobe directions (green and orange arrows in Fig. 4a), we carried out orientation-dependent STML measurements by moving the STM tip along an approximately circular trajectory around a single ZnPc molecule (red dashed line in Fig. 4a, representing the data points measured at *r*=0). The orientation angle of the tip position can be defined by setting the direction of one of these two dipoles as a reference direction with zero angle (*θ*=0).

Figure 4b shows two typical Fano spectra at zero detuning obtained for tip positions corresponding to orientation angles of 0° and 45°. The spectra appear to be almost overlapped, but a small frequency shift can be observed when the Fano dip region is enlarged (inset in Fig. 4b). Fig. 4c plots the Fano spectra as a function of tip positions for angles varying from 0° to 360° (one full round around the molecule), which reveals a clear periodical pattern with four periods corresponding to the four-lobe feature of the molecule. For polar angle positions of 0°, 90°, 180° and 270°, the tip is close to each of the four lobes of the ZnPc molecule, and the Fano dip (dark zone in the contour plot) exhibits a large Lamb shift (∼3 meV). While for tip positions with polar angles of 45°, 135°, 225° and 315°, a smaller Lamb shift is observed (∼1 meV). The orientation dependence of the Fano spectra for either positive or negative spectral detuning also exhibit a similar periodicity (Supplementary Note 7). The photonic Lamb shift is usually expressed in terms of the dyadic Green's function of the nanocavity (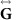) and the transition dipole moment of the molecule, namely, 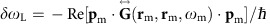
39. Thus an orientation dependence of the Lamb shift can be expected, although the explicit expression in terms of the Green's function is often difficult to obtain for a realistic nanocavity. Phenomenologically, we can approximate a functional dependence of the self-interaction induced Lamb shift on the orientation of the tip (*θ*) as cos^4^*θ* and sin^4^*θ* for individual molecular dipole moments 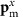 and 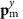, respectively (see Supplementary Note 7 for details). Following this approximation, the simulated Lamb shift would also exhibit the same periodicity for the Fano dip (dark zone in Fig. 4d), with a remarkable agreement of all the spectral features observed experimentally. The observation of strong orientation dependence of the Lamb shift, achieved by the fine spatial control in our experiment, demonstrates the anisotropic nature of the coherent coupling between the molecule and the nanocavity plasmon.

## Discussion

In conclusion, by using a single molecular emitter as a distinctive optical probe to coherently couple with the highly confined plasmonic nanocavity, we have demonstrated the Fano resonance and Lamb shift at the single-molecule level. The ability to spatially control the single-molecule Fano resonance with atomic precision allows to reveal the coherent and highly confined nature of the broadband nanocavity plasmon, as well as the coupling strength and the anisotropy of the field–matter interaction, providing new insights into the physical mechanism of the coherent coupling beyond the dipolar coupling model. Our findings open up new routes to probe and control quantum interference and field–matter interaction down to sub-nanometre scale, which could bring quantum photonic technologies down to the realm of nanoscale devices for single-molecule sensing, nano-optical modulation and quantum information processing.

## Methods

### Sample and tip preparation

ZnPc molecules were thermally evaporated onto the Ag(100) substrate partially covered by NaCl islands at about 8 K. The Ag(100) substrate was previously cleaned by cycles of argon ion sputtering and annealing. Electrochemically etched silver (Ag) tips used in all the experiments were cleaned in ultrahigh vacuum by outgassing and argon-ion sputtering. The desired NCP modes with different frequency detunings with respect to the molecular transition frequency were achieved by further modification of the tip morphology via voltage pulses or tip indentation on the bare Ag(100) surface.

### STML measurements

Our experiments were performed with a custom low-temperature ultrahigh-vacuum STM (Unisoku) equipped with photon collection and detection units at about 8 K under a base pressure of about 1 × 10^−10^ Torr. Topograph imaging and STML measurements were taken in a constant-current mode with the sample biased. Photons emitted from the STM junction were collected by a two-channel double-lens system to increase the collection efficiency^24^. The total hemisphere photon collection efficiency for the two-channel double-lens system is about 20%. The spectra were measured with a liquid-nitrogen-cooled charge-coupled device spectrometer (Princeton Instruments). Different gratings (600 grooves per mm and 1,200 grooves per mm) with a slit size of 100 μm were used in spectral measurements for different requirements on wavelength ranges and spectral resolutions. All spectra presented in this paper are not corrected for the wavelength-dependent sensitivity of photon detection systems. The thermal drift of our current system at about 8 K is 0.1–0.2 nm h^−1^, negligible for all the spectral measurements presented in this work.

### Data availability

Data available upon request from the authors.

## Additional information

**How to cite this article:** Zhang, Y. *et al*. Sub-nanometre control of the coherent interaction between a single molecule and a plasmonic nanocavity. *Nat. Commun.* **8,** 15225 doi: 10.1038/ncomms15225 (2017).

**Publisher's note:** Springer Nature remains neutral with regard to jurisdictional claims in published maps and institutional affiliations.

## Supplementary Material

Supplementary Information Supplementary Figures, Supplementary Notes and Supplementary References
