# Supplementary material for: Sub-nanometre control of the coherent interaction between a single molecule and a plasmonic nanocavity
Source: Nat Commun. 2017 May 19;8:15225. doi: 10.1038/ncomms15225 (PMC5454454; doi:10.1038/ncomms15225)
Supplement: Supplementary Information — Supplementary Figures, Supplementary Notes and Supplementary References [file ncomms15225-s1.pdf]

SUPPLEMENTARY INFORMATION

Supplementary Note 1. Definition of the tip position reference (*r*=0)

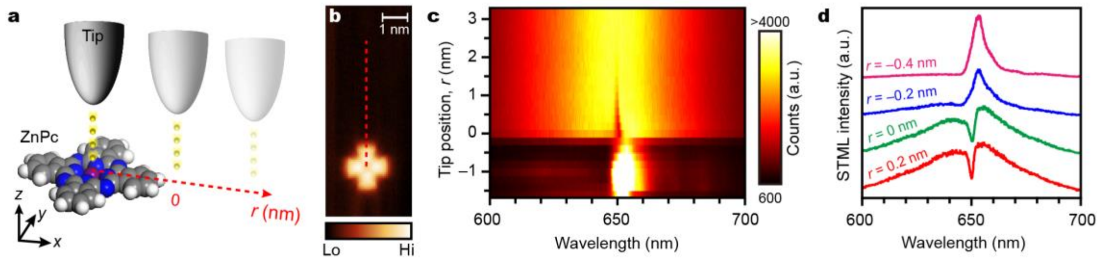

Supplementary Figure 1 | Definition of the tip position reference (*r*=0). a, Schematics showing the tip trajectory of STML measurements from the ZnPc molecule to the NaCl/Ag surface, marking the position reference defined for *r*=0 at the edge of the ZnPc molecule. **b**, STM image (−1.7 V, 2 pA) showing a line trace corresponding to the schematic tip trajectory in **a**. **c**, Color plot of a series of corresponding STML spectra (−2.5 V, 200 pA, 20 s) with a step size of 0.2 nm along the line trace in **b**. The zero reference point of the tip position is defined according to the abrupt change of the STML spectral feature from a Fano dip to a typical molecular fluorescence peak, as detailed in **d**. **d**, Representative STML spectra around the tip position reference (*r*=0), showing the abrupt change of the emission spectral lineshape.

By comparing the fluorescence and Fano spectra in Supplementary Fig. 1c and d, it can be seen that the plasmonic background in the fluorescence spectra decreases dramatically when the STM tip is located on the top of the ZnPc molecule, because in such a situation most of tunneling electrons directly excite the molecule, rather than the nanocavity plasmon.[1] Indeed, when the tip is above the molecule, a higher photonic local density of states might be expected, however, its role in this situation is merely to enhance the molecular radiative decay rate, known as the Purcell effect.

Supplementary Note 2. Coupled harmonic oscillator model

Before describing the coupling model between the plasmon and a single molecule, we would like to give a brief introduction on the local electric field (or light emission) in the STM junction excited by the tunneling electrons because in this work it is the tunneling current that acts as a source for the excitation of the nanocavity plasmon. Plasmonic emission is known to originate from the fluctuation of the tunneling current with the frequency dependent current density expressed as[2,3]

$$\mathbf{j}(\mathbf{r},\omega) = j_0 \hat{\mathbf{u}}_z \sqrt{\frac{1-\frac{\hbar\omega}{eV}}{1-e^{-\frac{eV}{kT}\left(1-\frac{\hbar\omega}{eV}\right)}}}\,\delta(\mathbf{r}-\mathbf{r}_0), \quad (1)$$

in which V is the applied voltage, e the electron charge, $\hbar$ the reduced Planck constant, k the Boltzmann constant and T the temperature. $\delta(\mathbf{r}-\mathbf{r}_0)$ is the delta function at the tip position $\mathbf{r}_0$, which describes the highly localized nature of the tunneling current. $\mathbf{u}_z$ is the unit vector along the z-axis perpendicular to the surface of substrate, and j_0 is the applied DC tunneling current density. The electric field induced by this tunneling current can be obtained as[4]

$$\mathbf{E}_\mathrm{j}(\mathbf{r},\omega) = \frac{i}{\omega}\int \overleftrightarrow{\mathbf{G}}_0(\mathbf{r},\mathbf{r}';\omega)\,\mathbf{j}(\mathbf{r}',\omega)\,d^3r', \quad (2)$$

where $\overleftrightarrow{\mathbf{G}}_0$ is the dyadic Green's function in vacuum. (Equation 2 sets the definition of the Green function that we use, with other possible definitions in the literature affecting the multiplicative prefactors in the expressions.) For a nanocavity composed by a metallic tip and substrate, the resulted local plasmonic field from the tunneling current would be enhanced, a situation that can be considered by replacing the Green's

function in Eq. (2) by the Green's function in the presence of the plasmonic system $\overleftrightarrow{\mathbf{G}}_{\text{loc}}$. An enhancement factor $\overleftrightarrow{\mathbf{M}}$ can thus be defined from the following expression[5]

$$\mathbf{E}_{\text{loc}}(\mathbf{r},\omega)=\frac{i}{\omega}\int\overleftrightarrow{\mathbf{G}}_{\text{loc}}(\mathbf{r},\mathbf{r}';\omega)\,\mathbf{j}(\mathbf{r}',\omega)\,d^3r'\equiv\overleftrightarrow{\mathbf{M}}(\mathbf{r},\mathbf{r}_0;\omega)\,\mathbf{E}_{\mathbf{j}}(\mathbf{r}_0,\omega). \quad (3)$$

If we treat the current-excited nanocavity plasmon as an effective dipole moment $\mathbf{p}_{\text{p}}$ due to the polarization of the field ($\mathbf{p}_{\text{p}}=\alpha_{\text{p}}\mathbf{E}_{\mathbf{j}}(\mathbf{r}_{\text{p}},\omega)$), for practical purpose, the total enhanced field associated with this plasmonic dipole can also be expressed as

$$\mathbf{E}_{\text{loc}}(\mathbf{r},\omega)=\overleftrightarrow{\mathbf{G}}_0(\mathbf{r},\mathbf{r}_{\text{p}};\omega)\mathbf{p}_{\text{p}}=\overleftrightarrow{\mathbf{G}}_0(\mathbf{r},\mathbf{r}_{\text{p}};\omega)\alpha_{\text{p}}\mathbf{E}_{\mathbf{j}}(\mathbf{r}_{\text{p}},\omega)\equiv\overleftrightarrow{\mathbf{M}}(\mathbf{r},\mathbf{r}_{\text{p}};\omega)\mathbf{E}_{\mathbf{j}}(\mathbf{r}_{\text{p}},\omega). \quad (4)$$

Thus the local electric field induced by the tunneling current can serve as a local source of excitation. For simplicity, due to the mild variation of $\mathbf{j}(\mathbf{r}, \omega)$ for frequencies smaller than the bias, the electric field $\mathbf{E}_{\mathbf{j}}$ is assumed to be constant here.

We now proceed to consider the interaction of a single molecule with the nanocavity plasmon, treating both as a harmonic oscillator, namely, within the framework of dipole approximation. If a single molecule is placed in the nanocavity, the molecular electronic transition would be excited by the nanocavity plasmon that is previously excited by the tunneling current. This molecular transition dipole moment $\mathbf{p}_{\text{m}}$ would in turn interact with the nanocavity plasmon. The whole interaction process between the molecular transition dipole moment ($\mathbf{p}_{\text{m}}$) and plasmonic effective dipole moment ($\mathbf{p}_{\text{p}}$) can be expressed by the following coupled equations

$$\begin{cases}\mathbf{p}_{\text{m}}=\alpha_{\text{m}}\mathbf{E}_{\text{loc}}=\alpha_{\text{m}}\left[\overleftrightarrow{\mathbf{G}}_0(\mathbf{r}_{\text{m}},\mathbf{r}_{\text{p}};\omega)\cdot\mathbf{p}_{\text{p}}\right]\\ \mathbf{p}_{\text{p}}=\alpha_{\text{p}}\left[\mathbf{E}_{\mathbf{j}}(\mathbf{r}_{\text{p}},\omega)+\overleftrightarrow{\mathbf{G}}_0(\mathbf{r}_{\text{p}},\mathbf{r}_{\text{m}};\omega)\cdot\mathbf{p}_{\text{m}}\right]\end{cases}, \quad (5)$$

in which α_{m} and α_{p} are the polarizabilities of molecular and plasmonic dipole moments, respectively. The interaction terms are described by the corresponding Green's

functions of $\mathbf{p}_m$ and $\mathbf{p}_p$, respectively. By defining $\overleftrightarrow{\mathbf{G}}^{\mathrm{d}}_{\mathrm{mp}} \equiv \overleftrightarrow{\mathbf{G}}_0\left(\mathbf{r}_\mathrm{m},\mathbf{r}_\mathrm{p};\omega\right)$ and $\overleftrightarrow{\mathbf{G}}^{\mathrm{d}}_{\mathrm{pm}} \equiv \overleftrightarrow{\mathbf{G}}_0\left(\mathbf{r}_\mathrm{p},\mathbf{r}_\mathrm{m};\omega\right)$, Eq. (5) can be simplified as

$$\begin{cases} \mathbf{p}_\mathrm{m} = \alpha_\mathrm{m}\overleftrightarrow{\mathbf{G}}^{\mathrm{d}}_{\mathrm{mp}}\cdot\mathbf{p}_\mathrm{p} \\ \mathbf{p}_\mathrm{p} = \alpha_\mathrm{p}\left(\mathbf{E}_\mathbf{j}+\overleftrightarrow{\mathbf{G}}^{\mathrm{d}}_{\mathrm{pm}}\cdot\mathbf{p}_\mathrm{m}\right) \end{cases}. \tag{6}$$

The solution for the plasmonic dipole $\mathbf{p}_p$ can be obtained as

$$\mathbf{p}_\mathrm{p} = \alpha_\mathrm{p}\left(\overleftrightarrow{\mathbf{I}}+\frac{\overleftrightarrow{\mathbf{G}}^{\mathrm{d}}_{\mathrm{mp}}\alpha_\mathrm{p}\overleftrightarrow{\mathbf{G}}^{\mathrm{d}}_{\mathrm{pm}}}{\alpha_\mathrm{m}^{-1}-\overleftrightarrow{\mathbf{G}}^{\mathrm{d}}_{\mathrm{mp}}\alpha_\mathrm{p}\overleftrightarrow{\mathbf{G}}^{\mathrm{d}}_{\mathrm{pm}}}\right)\mathbf{E}_\mathbf{j}, \tag{7}$$

from which we can define a self-interaction term of the molecular dipole as $\overleftrightarrow{\mathbf{G}}^{\mathrm{d}}_{\mathrm{mm}} \equiv \overleftrightarrow{\mathbf{G}}^{\mathrm{d}}_{\mathrm{mp}}\alpha_\mathrm{p}\overleftrightarrow{\mathbf{G}}^{\mathrm{d}}_{\mathrm{pm}}$ and use such notation in the following. The polarizabilities of the molecule and plasmon are considered to be isotropic and can be expressed as

$$\alpha_\mathrm{m} = \alpha_\mathrm{m}^0\frac{\omega_\mathrm{m}^2}{\omega_\mathrm{m}^2-\omega^2-i\omega\gamma_\mathrm{m}^0}, \tag{8}$$

$$\alpha_\mathrm{p} = \alpha_\mathrm{p}^0\frac{\omega_\mathrm{p}^2}{\omega_\mathrm{p}^2-\omega^2-i\omega\gamma_\mathrm{p}}. \tag{9}$$

Here ω_m is the transition frequency of the molecule, ω_p is the resonant frequency of the plasmon, $\alpha^0_{\mathrm{m(p)}}$ is defined as the static polarizability of the molecule (plasmon), and γ_m^0 (γ_p) corresponds to the total losses of the molecule (plasmon). The emission spectrum in terms of radiative power can then be obtained as[4]

$$P(\omega) = \frac{\omega^4}{12\pi\varepsilon_0 c^3}\left|\mathbf{p}_\mathrm{p}\right|^2. \tag{10}$$

From Eqs. (7−9), the above equation for the emission spectrum can be re-written into a typical expression of the Fano lineshape:

$$
\begin{aligned}
P(\omega) &= \frac{\omega^4 \left|\alpha_{\mathrm{p}}\mathbf{E}_{\mathbf{j}}\right|^2}{12\pi\varepsilon_0 c^3}\left|\frac{\omega_{\mathrm{m}}^2-\omega^2-i\omega\gamma_{\mathrm{m}}^0}{\omega_{\mathrm{m}}^2-\omega^2-i\omega\gamma_{\mathrm{m}}^0-\alpha_{\mathrm{m}}^0\omega_{\mathrm{m}}^2 G_{\mathrm{mm}}^{\mathrm{d}}}\right|^2 \\
&\approx \frac{\omega^4 \left|\alpha_{\mathrm{p}}\mathbf{E}_{\mathbf{j}}\right|^2}{12\pi\varepsilon_0 c^3}\frac{\left[\dfrac{\omega_{\mathrm{m}}^2-\omega^2-\alpha_{\mathrm{m}}^0\omega_{\mathrm{m}}^2\,\mathrm{Re}\left[G_{\mathrm{mm}}^{\mathrm{d}}\right]}{\omega\gamma_{\mathrm{m}}^0+\alpha_{\mathrm{m}}^0\omega_{\mathrm{m}}^2\,\mathrm{Im}\left[G_{\mathrm{mm}}^{\mathrm{d}}\right]}+\dfrac{\alpha_{\mathrm{m}}^0\omega_{\mathrm{m}}^2\,\mathrm{Re}\left[G_{\mathrm{mm}}^{\mathrm{d}}\right]}{\omega\gamma_{\mathrm{m}}^0+\alpha_{\mathrm{m}}^0\omega_{\mathrm{m}}^2\,\mathrm{Im}\left[G_{\mathrm{mm}}^{\mathrm{d}}\right]}\right]^2+\left(\dfrac{\omega\gamma_{\mathrm{m}}^0}{\omega\gamma_{\mathrm{m}}^0+\alpha_{\mathrm{m}}^0\omega_{\mathrm{m}}^2\,\mathrm{Im}\left[G_{\mathrm{mm}}^{\mathrm{d}}\right]}\right)^2}{\left[\dfrac{\omega_{\mathrm{m}}^2-\omega^2-\alpha_{\mathrm{m}}^0\omega_{\mathrm{m}}^2\,\mathrm{Re}\left[G_{\mathrm{mm}}^{\mathrm{d}}\right]}{\omega\gamma_{\mathrm{m}}^0+\alpha_{\mathrm{m}}^0\omega_{\mathrm{m}}^2\,\mathrm{Im}\left[G_{\mathrm{mm}}^{\mathrm{d}}\right]}\right]^2+1} \\
&\equiv A\frac{(\Omega+q)^2+B}{\Omega^2+1}.
\end{aligned}
\tag{11}
$$

Here the dimensionless frequency Ω is defined as $\Omega \equiv -\left(\omega_{\mathrm{m}}^2-\omega^2-\alpha_{\mathrm{m}}^0\omega_{\mathrm{m}}^2\,\mathrm{Re}\left[G_{\mathrm{mm}}^{\mathrm{d}}\right]\right)/\left(\omega\gamma_{\mathrm{m}}^0+\alpha_{\mathrm{m}}^0\omega_{\mathrm{m}}^2\,\mathrm{Im}\left[G_{\mathrm{mm}}^{\mathrm{d}}\right]\right)$; $A \equiv \left(\omega^4\left|\alpha_{\mathrm{p}}\mathbf{E}_{\mathbf{j}}\right|^2\right)/\left(12\pi\varepsilon_0 c^3\right)$, which can be regarded as the background of the spectrum; $B \equiv \left[\omega\gamma_{\mathrm{m}}^0/\left(\omega\gamma_{\mathrm{m}}^0+\alpha_{\mathrm{m}}^0\omega_{\mathrm{m}}^2\,\mathrm{Im}\left[G_{\mathrm{mm}}^{\mathrm{d}}\right]\right)\right]^2$, which ensures the Fano dip depth to be non-zero for the condition $\Omega+q=0$. q is the so-called Fano parameter, which reflects the symmetry properties of the Fano lineshape and is defined as

$$
q \equiv -\frac{\alpha_{\mathrm{m}}^0\omega_{\mathrm{m}}^2\,\mathrm{Re}\left[G_{\mathrm{mm}}^{\mathrm{d}}\right]}{\omega\gamma_{\mathrm{m}}^0+\alpha_{\mathrm{m}}^0\omega_{\mathrm{m}}^2\,\mathrm{Im}\left[G_{\mathrm{mm}}^{\mathrm{d}}\right]}. \tag{12}
$$

According to Eq. (11), if there is no interaction between the molecule and the plasmon (i.e., $G_{\mathrm{mm}}^{\mathrm{d}}=0$, as a result of $G_{\mathrm{mp}}^{\mathrm{d}}=G_{\mathrm{pm}}^{\mathrm{d}}=0$), $P(\omega)$ would be proportional to $|\alpha_{\mathrm{p}}\mathbf{E}_{\mathbf{j}}|^2$, giving rise to a plasmonic emission spectrum characterized by a Lorentzian lineshape. When there is significant interaction, a “dip”-like feature around the molecular transition frequency would appear, known as the Fano lineshape (as shown in Supplementary Figs. 2a–c). The dip position is determined by the condition $\Omega+q=0$, yielding $\omega_{\mathrm{dip}}=\omega_{\mathrm{m}}$ within the dipole model.

It should be noted that for zero detuning condition ($\Delta \equiv \omega_{\mathrm{p}}-\omega_{\mathrm{m}}=0$), the polarizability of plasmonic dipole would be a pure imaginary number according to Eq. (9), thus the self-interaction term $G_{\mathrm{mm}}^{\mathrm{d}}=G_{\mathrm{mp}}^{\mathrm{d}}\alpha_{\mathrm{p}}G_{\mathrm{pm}}^{\mathrm{d}}$ would be a pure imaginary number since

$G^{\mathrm{d}}_{\mathrm{mp}}G^{\mathrm{d}}_{\mathrm{pm}}$ is real when using the expression of near-field Green's function for a dipole. As a result, a zero Fano parameter (q=0) is obtained according Eq. (12). In other words, the Fano lineshape would present a perfect symmetric dip (Supplementary Fig. 2b). For the positive detuning (Δ>0) and negative detuning (Δ<0), the Fano parameter q would be positive and negative, respectively, showing an asymmetric dip feature as schematically illustrated in Supplementary Figs. 2a and c. Notably, within the framework of the dipole approximation described here, the predicted Fano spectral features would reveal a "mirror-symmetry" for the same amount of positive and negative detunings, which does not properly describe the experimental observations.

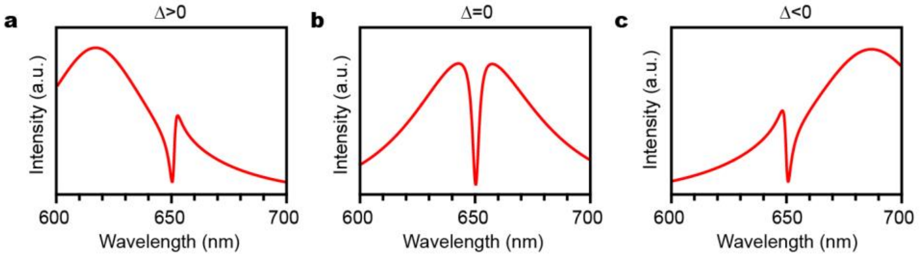

Supplementary Figure 2 | Typical Fano spectral feature in different detuning conditions according to the coupled harmonic oscillator model. a, Δ>0, **b**, Δ=0 and **c**, Δ<0.

Supplementary Note 3. Generalized coupled oscillator model

As shown in the main-text Figs. 2−4, experimental STML spectra reveal both the shift of the Fano dip positions and asymmetric Fano lineshapes, which cannot be explained by the coupled dipole model described above in Supplementary Note 2. To account for the experimental observations, a theoretical model beyond the dipole interaction can be considered. To this end, we start from Eq. (6) and rewrite it as

$$\begin{cases} \mathbf{p}_{\mathrm{m}} = \alpha_{\mathrm{m}} \left(\overleftrightarrow{\mathbf{G}}_{\mathrm{mp}} \alpha_{\mathrm{p}} \mathbf{E}_{\mathbf{j}} + \overleftrightarrow{\mathbf{G}}^{\mathrm{d}}_{\mathrm{mp}} \alpha_{\mathrm{p}} \overleftrightarrow{\mathbf{G}}^{\mathrm{d}}_{\mathrm{pm}} \cdot \mathbf{p}_{\mathrm{m}} \right) \\ \mathbf{p}_{\mathrm{p}} = \alpha_{\mathrm{p}} \left(\mathbf{E}_{\mathbf{j}} + \overleftrightarrow{\mathbf{G}}^{\mathrm{d}}_{\mathrm{pm}} \cdot \mathbf{p}_{\mathrm{m}} \right) \end{cases}. \tag{13}$$

By defining the field enhancement term $\overleftrightarrow{\mathbf{M}}^{\mathrm{d}} \equiv \overleftrightarrow{\mathbf{G}}^{\mathrm{d}}_{\mathrm{mp}} \alpha_{\mathrm{p}}$ and the self-interaction term of the molecular dipole $\overleftrightarrow{\mathbf{G}}^{\mathrm{d}}_{\mathrm{mm}} \equiv \overleftrightarrow{\mathbf{G}}^{\mathrm{d}}_{\mathrm{mp}} \alpha_{\mathrm{p}} \overleftrightarrow{\mathbf{G}}^{\mathrm{d}}_{\mathrm{pm}}$, we obtain

$$\begin{cases} \mathbf{p}_{\mathrm{m}} = \alpha_{\mathrm{m}} \left(\overleftrightarrow{\mathbf{M}}^{\mathrm{d}} \cdot \mathbf{E}_{\mathbf{j}} + \overleftrightarrow{\mathbf{G}}^{\mathrm{d}}_{\mathrm{mm}} \cdot \mathbf{p}_{\mathrm{m}} \right) \\ \mathbf{p}_{\mathrm{p}} = \alpha_{\mathrm{p}} \left(\mathbf{E}_{\mathbf{j}} + \overleftrightarrow{\mathbf{G}}^{\mathrm{d}}_{\mathrm{pm}} \cdot \mathbf{p}_{\mathrm{m}} \right) \end{cases}. \tag{14}$$

As we have shown in Supplementary Note 2, the model based on the consideration of dipole interaction alone ($\overleftrightarrow{\mathbf{M}}^{\mathrm{d}}$ and $\overleftrightarrow{\mathbf{G}}^{\mathrm{d}}_{\mathrm{mm}}$ are only related with $\overleftrightarrow{\mathbf{G}}^{\mathrm{d}}_{\mathrm{mp}}$ and α_{p}) cannot fully explain the experimental observations. Therefore, we generalize the model by replacing the dipole-interaction based $\overleftrightarrow{\mathbf{M}}^{\mathrm{d}}$, $\overleftrightarrow{\mathbf{G}}^{\mathrm{d}}_{\mathrm{mm}}$ and $\overleftrightarrow{\mathbf{G}}^{\mathrm{d}}_{\mathrm{pm}}$ in Eq. (14) with the full-interaction based $\overleftrightarrow{\mathbf{M}}$, $\overleftrightarrow{\mathbf{G}}_{\mathrm{mm}}$ and $\overleftrightarrow{\mathbf{G}}_{\mathrm{pm}}$ to account for additional contributions such as higher order plasmonic modes and the spatial distribution of the molecular transition dipole, yielding generalized coupling equations as follows:

$$\begin{cases} \mathbf{p}_{\mathrm{m}} = \alpha_{\mathrm{m}} \left(\overleftrightarrow{\mathbf{M}} \cdot \mathbf{E}_{\mathbf{j}} + \overleftrightarrow{\mathbf{G}}_{\mathrm{mm}} \cdot \mathbf{p}_{\mathrm{m}} \right) \\ \mathbf{p}_{\mathrm{p}} = \alpha_{\mathrm{p}} \left(\mathbf{E}_{\mathbf{j}} + \overleftrightarrow{\mathbf{G}}_{\mathrm{pm}} \cdot \mathbf{p}_{\mathrm{m}} \right) \end{cases}. \tag{15}$$

A generalized solution for the plasmonic dipole is

$$\mathbf{p}_{\mathrm{p}} = \alpha_{\mathrm{p}} \left(\overleftrightarrow{\mathbf{I}} + \frac{\overleftrightarrow{\mathbf{G}}_{\mathrm{pm}} \cdot \overleftrightarrow{\mathbf{M}}}{\alpha_{\mathrm{m}}^{-1} - \overleftrightarrow{\mathbf{G}}_{\mathrm{mm}}} \right) \mathbf{E}_{\mathbf{j}}. \tag{16}$$

This expression, although similar to Eq. (7), takes into account the contributions from the higher-order interactions and the spatial distribution of the molecular transition dipole beyond the dipole approximation. If we define a residual self-interaction term as $\overleftrightarrow{\mathbf{G}}^{\mathrm{res}}_{\mathrm{mm}} = \overleftrightarrow{\mathbf{G}}_{\mathrm{mm}} - \overleftrightarrow{\mathbf{G}}_{\mathrm{pm}} \cdot \overleftrightarrow{\mathbf{M}}$ (which would be zero within the dipole approximation) to

account for the non-dipole contributions, and adopt Eq. (8) to describe the molecular polarizability α_{m}, the plasmonic dipole can then be expressed as

$$\begin{aligned}\mathbf{p}_{\mathrm{p}} &= \alpha_{\mathrm{p}}\left[\overleftrightarrow{\mathbf{I}}+\frac{\alpha_{\mathrm{m}}^{0}\omega_{\mathrm{m}}^{2}\overleftrightarrow{\mathbf{G}}_{\mathrm{pm}}\cdot\overleftrightarrow{\mathbf{M}}}{\left(\omega_{\mathrm{m}}^{2}-\omega^{2}-i\omega\gamma_{\mathrm{m}}^{0}\right)-\alpha_{\mathrm{m}}^{0}\omega_{\mathrm{m}}^{2}\overleftrightarrow{\mathbf{G}}_{\mathrm{mm}}}\right]\mathbf{E}_{\mathbf{j}} \\ &= \alpha_{\mathrm{p}}\left[\frac{\left(\omega_{\mathrm{m}}^{2}-\omega^{2}-i\omega\gamma_{\mathrm{m}}^{0}\right)-\alpha_{\mathrm{m}}^{0}\omega_{\mathrm{m}}^{2}\overleftrightarrow{\mathbf{G}}_{\mathrm{mm}}^{\mathrm{res}}}{\left(\omega_{\mathrm{m}}^{2}-\omega^{2}-i\omega\gamma_{\mathrm{m}}^{0}\right)-\alpha_{\mathrm{m}}^{0}\omega_{\mathrm{m}}^{2}\overleftrightarrow{\mathbf{G}}_{\mathrm{mm}}}\right]\mathbf{E}_{\mathbf{j}}\end{aligned}. \quad (17)$$

We now define the following notations:

$$\delta\omega_{\mathrm{L}}' = -\frac{\omega_{\mathrm{m}}}{2}\alpha_{\mathrm{m}}^{0}\,\mathrm{Re}\left[\hat{\mathbf{n}}_{\mathrm{m}}\cdot\overleftrightarrow{\mathbf{G}}_{\mathrm{mm}}^{\mathrm{res}}\cdot\hat{\mathbf{n}}_{\mathrm{m}}\right], \quad (18)$$

$$\gamma_{\mathrm{m}}' = \gamma_{\mathrm{m}}^{0}+\alpha_{\mathrm{m}}^{0}\omega_{\mathrm{m}}\,\mathrm{Im}\left[\mathbf{n}_{\mathrm{m}}\cdot\overleftrightarrow{\mathbf{G}}_{\mathrm{mm}}^{\mathrm{res}}\cdot\mathbf{n}_{\mathrm{m}}\right]\equiv\gamma_{\mathrm{m}}^{0}+F_{\mathrm{P}}^{\mathrm{res}}\gamma_{\mathrm{m}}^{\mathrm{s}}, \quad (19)$$

$$\delta\omega_{\mathrm{L}} = -\frac{\omega_{\mathrm{m}}}{2}\alpha_{\mathrm{m}}^{0}\,\mathrm{Re}\left[\hat{\mathbf{n}}_{\mathrm{m}}\cdot\overleftrightarrow{\mathbf{G}}_{\mathrm{mm}}\cdot\hat{\mathbf{n}}_{\mathrm{m}}\right], \quad (20)$$

$$\gamma_{\mathrm{m}} = \gamma_{\mathrm{m}}^{0}+\alpha_{\mathrm{m}}^{0}\omega_{\mathrm{m}}\,\mathrm{Im}\left[\mathbf{n}_{\mathrm{m}}\cdot\overleftrightarrow{\mathbf{G}}_{\mathrm{mm}}\cdot\mathbf{n}_{\mathrm{m}}\right]\equiv\gamma_{\mathrm{m}}^{0}+F_{\mathrm{P}}\gamma_{\mathrm{m}}^{\mathrm{s}}, \quad (21)$$

where F_{P} and $F_{\mathrm{P}}^{\mathrm{res}}$ are the Purcell factors corresponding to the total and residual self-interaction, respectively. $\gamma_{\mathrm{m}}^{\mathrm{s}}$ is the intrinsic spontaneous decay rate of the molecule. (For a single ZnPc molecule considered here, the transition dipole moment is about 10.6 Debye,[1,6] corresponding to a spontaneous decay rate $\gamma_{\mathrm{m}}^{\mathrm{s}}\approx 8.41\times10^{-5}$ meV, much weaker than other decay or dephasing channels.[7,8]) The Purcell factor is thus defined here as the plasmonic enhancement of the spontaneous decay rate.

Notably, $\delta\omega_{\mathrm{L}}$ can be assigned as the Lamb shift which is related to the change of the molecular transition frequency due to the self-interaction of the molecule via the surrounding environment. Such assignment can be justified as follows. If we consider the quantum expression for α_{m}^{0} according to the literature (e.g., Ref. [4])

$$\alpha_{\mathrm{m}}^{0} = \frac{e^{2}}{m_{e}\omega_{\mathrm{m}}^{2}}\overleftrightarrow{\mathbf{f}}_{\mathrm{m}} = \frac{e^{2}}{m_{e}\omega_{\mathrm{m}}^{2}}\frac{2m_{e}\omega_{\mathrm{m}}}{e^{2}\hbar}\mathbf{p}_{\mathrm{m}}^{0}\mathbf{p}_{\mathrm{m}}^{0} = \frac{2}{\hbar\omega_{\mathrm{m}}}\mathbf{p}_{\mathrm{m}}^{0}\mathbf{p}_{\mathrm{m}}^{0}, \quad (22)$$

where $\overleftrightarrow{\mathbf{f}}_{\mathrm{m}}$ is the oscillator strength, and $\mathbf{p}_{\mathrm{m}}^{0}\mathbf{p}_{\mathrm{m}}^{0}$ is the matrix formed by the out product between the molecular transition dipole moments, and insert Eq. (22) into Eq. (20), $\delta\omega_{\mathrm{L}}$ can then be expressed as

$$\delta\omega_{\mathrm{L}} = -\frac{1}{\hbar}\mathrm{Re}\left[\mathbf{p}_{\mathrm{m}}^{0}\cdot\overleftrightarrow{\mathbf{G}}_{\mathrm{mm}}\cdot\mathbf{p}_{\mathrm{m}}^{0}\right], \tag{23}$$

which is identical to the standard form of Lamb shift.[9]

Using the defined notations from Eqs. (18) to (21), the solution of Eq. (17) can be approximated as

$$\mathbf{p}_{\mathrm{p}} = \alpha_{\mathrm{p}}\mathbf{E}_{\mathbf{j}}\left[\frac{\left(\omega_{\mathrm{m}}+\delta\omega_{\mathrm{L}}'\right)^{2}-\omega^{2}-i\omega\gamma_{\mathrm{m}}'}{\left(\omega_{\mathrm{m}}+\delta\omega_{\mathrm{L}}\right)^{2}-\omega^{2}-i\omega\gamma_{\mathrm{m}}}\right]. \tag{24}$$

This expression can be also used to derive the standard description of the Fano lineshape in terms of the q-parameter as:

$$\begin{aligned} P(\omega) &= \frac{\omega^{4}\left|\alpha_{\mathrm{p}}\mathbf{E}_{\mathbf{j}}\right|^{2}}{12\pi\varepsilon_{0}c^{3}}\left|\frac{\left(\omega_{\mathrm{m}}+\delta\omega_{\mathrm{L}}'\right)^{2}-\omega^{2}-i\omega\gamma_{\mathrm{m}}'}{\left(\omega_{\mathrm{m}}+\delta\omega_{\mathrm{L}}\right)^{2}-\omega^{2}-i\omega\gamma_{\mathrm{m}}}\right|^{2} \\ &\approx \frac{\omega^{4}\left|\alpha_{\mathrm{p}}\mathbf{E}_{\mathbf{j}}\right|^{2}}{12\pi\varepsilon_{0}c^{3}}\frac{\left[\frac{\omega^{2}-\left(\omega_{\mathrm{m}}+\delta\omega_{\mathrm{L}}\right)^{2}}{\omega\gamma_{\mathrm{m}}}+\frac{2\left(\delta\omega_{\mathrm{L}}-\delta\omega_{\mathrm{L}}'\right)}{\gamma_{\mathrm{m}}}\right]^{2}+\left(\frac{\gamma_{\mathrm{m}}'}{\gamma_{\mathrm{m}}}\right)^{2}}{\left[\frac{\omega^{2}-\left(\omega_{\mathrm{m}}+\delta\omega_{\mathrm{L}}\right)^{2}}{\omega\gamma_{\mathrm{m}}}\right]^{2}+1} \\ &\equiv A\frac{\left(\Omega+q\right)^{2}+B}{\Omega^{2}+1}. \end{aligned} \tag{25}$$

It can be found that Eq. (25) gives the same expression as in Eq. (11), although Ω, q, A and B are related to a different set of parameters in this case. Particularly, for the Fano parameter q we now have

$$q = \frac{2\left(\delta\omega_{\mathrm{L}}-\delta\omega_{\mathrm{L}}'\right)}{\gamma_{\mathrm{m}}}. \tag{26}$$

As we have mentioned above, within the dipole approximation in Supplementary Note 2, $\overleftrightarrow{\mathbf{G}}^{\mathrm{res}}_{\mathrm{mm}}$ would be zero and $\overleftrightarrow{\mathbf{G}}_{\mathrm{mm}}$ would be a pure imaginary number, giving a zero value of the Fano parameter q in a situation of zero detuning (i.e., a symmetric dip in Supplementary Fig. 2b). For the generalized model that includes higher order interactions and spatial distribution of the molecular transition dipole, Eq. (26) properly accounts for the subtle asymmetry of the Fano profile found in the experiments. As shown in Supplementary Fig. 3b, an asymmetric Fano dip can be obtained by this generalized model for the zero-detuning condition. While for the positive detuning (Δ>0) and negative detuning (Δ<0), the corresponding Fano spectra, as shown in Supplementary Figs. 3a and c, are no longer of "mirror-symmetry", different from those in Supplementary Figs. 2a and c. The Fano dip position can also be estimated from the condition Ω + q = 0 in Eq. (25), giving $\omega_{\mathrm{dip}} = \omega_{\mathrm{m}} + \delta\omega'_{\mathrm{L}}$. The extra term $\delta\omega'_{\mathrm{L}}$ accounts for the dip shift, which would be zero in previous dipole model, where $\omega_{\mathrm{dip}} = \omega_{\mathrm{m}}$. We discuss in more detail the behavior and origin of the asymmetry in Supplementary Note 6.

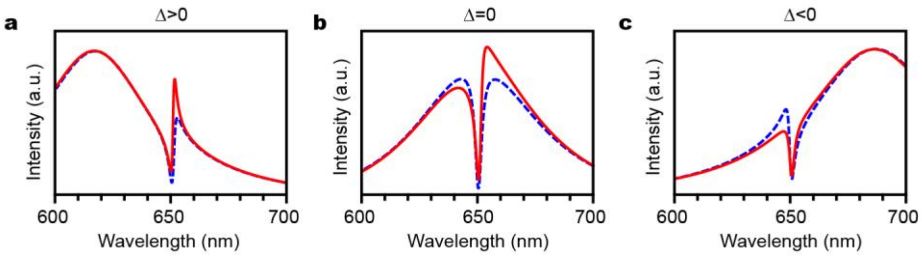

Supplementary Figure 3 | Typical Fano spectral feature from the generalized coupled oscillator model. a–c, Typical Fano spectra (red lines) for different detuning conditions: Δ>0 (**a**), Δ=0 (**b**) and Δ<0 (**c**), respectively. For comparison, the Fano spectra calculated from the dipole model described in Supplementary Note 2 (same

spectra as in Supplementary Fig. 2) are also plotted as the blue dashed curves. Notice the extra asymmetry in the spectra induced by contributions to the interaction beyond the dipole approximation.

Supplementary Note 4. Expressions for the normalized dip depth (D_{norm}) and effective coupling strength (g)

For the molecular emission in the plasmonic nanocavity, we can define an effective Purcell factor F_{P}' associated with the dipolar contribution to the total Purcell factor as follows:

$$F_{\mathrm{P}}' \equiv F_{\mathrm{P}} - F_{\mathrm{P}}^{\mathrm{res}} = \frac{\gamma_{\mathrm{m}} - \gamma_{\mathrm{m}}'}{\gamma_{\mathrm{m}}^{\mathrm{s}}}, \tag{27}$$

where $F_{\mathrm{P}}^{\mathrm{res}}$ and F_{P} are given by Eqs. (19) and (21), respectively. On the other hand, the effective Purcell factor can be related to an effective coupling strength g, as[10]

$$F_{\mathrm{P}}' = \frac{4g^2}{\gamma_{\mathrm{p}}\gamma_{\mathrm{m}}^{\mathrm{s}}}. \tag{28}$$

Combining Eq. (28) with Eq. (27) we obtain

$$g = \sqrt{\frac{\gamma_{\mathrm{p}}\left(\gamma_{\mathrm{m}} - \gamma_{\mathrm{m}}'\right)}{4}}. \tag{29}$$

Furthermore, we can define the normalized Fano dip depth as

$$D_{\mathrm{norm}} = 1 - \left|\mathbf{p}_{\mathrm{p}}\right|^2 / \left|\mathbf{p}_{\mathrm{p}}^0\right|^2 \Big|_{\omega=\omega_{\mathrm{dip}}}, \tag{30}$$

where $\mathbf{p}_{\mathrm{p}}^0 = \alpha_{\mathrm{p}}\mathbf{E}_{\mathrm{j}}$ denotes the plasmon dipole moment in the absence of the molecule, which is associated to the plasmonic background spectra used for normalization. The

normalized dip depth $D_{\rm norm}$ is evaluated at $\omega=\omega_{\rm dip}$, and can be obtained from Eq. (25) as

$$D_{\rm norm} \approx 1-\left|\frac{\gamma'_{\rm m}}{\gamma_{\rm m}}\right|^2 . \tag{31}$$

By combining Eqs. (29) and (31), we obtain

$$D_{\rm norm} \approx 1-\left|\frac{\gamma_{\rm p}\gamma'_{\rm m}}{4g^2+\gamma_{\rm p}\gamma'_{\rm m}}\right|^2 . \tag{32}$$

Therefore, the effective coupling strength g can be expressed in terms of the normalized Fano dip depth as

$$g \approx \sqrt{\frac{\gamma_{\rm p}\gamma'_{\rm m}}{4}\left(\sqrt{\frac{1}{1-D_{\rm norm}}}-1\right)} . \tag{33}$$

Supplementary Fig. 4a shows the normalized Fano spectra observed experimentally, with the normalized Fano dip depth indicated by an arrow between the dashed lines. A good fit can be obtained using Eq. (25) with fitting parameters $\delta\omega'_{\rm L}$, $\gamma'_{\rm m}$, $\delta\omega_{\rm L}$ and $\gamma_{\rm m}$, as shown in Supplementary Fig. 4b. The effective coupling strength g can be estimated from Eq. (33), whose variations with the tip position r are plotted in Supplementary Fig. 4c. The exponential fit of the decay of the coupling strength g exhibits an effective interaction distance within ~1 nm, which is consistent with the values obtained in main-text Fig. 2d based on the variation of normalized dip depth. It should be noted that the coupling strength g can be considered to be proportional to $|\mathbf{p}_{\rm m}\cdot\mathbf{E}_{\rm loc}|$; the fast exponential decay of the coupling strength g suggests that the effective interaction distance of the plasmonic field is highly confined within ~1 nm. From Eq. (28), we obtain a relatively large effective Purcell factor of 6.9×10^4 for a coupling strength g=15 meV obtained from the analysis of experimental Fano spectra.

Nevertheless, if we deduce the Purcell factor from the cavity parameters themselves via $F_P=[3\lambda^3/(4\pi^2)](Q/V)$, a much larger value of 1.7×10^6 can be obtained for a plasmonic nanocavity with the resonance wavelength λ=650 nm, the quality factor $Q=\omega_p/\gamma_p\approx12$, and the mode volume V~150 nm^3 (estimated by finite element methods for a cavity composed of a Ag sphere with a radius of R=10 nm and a Ag substrate with a gap distance of 1.5 nm). The difference between one and two orders of magnitude can be related to the differences in the molecular position and orientation inside the nanocavity. In our case, the molecule is located at a certain distance away from the center of the cavity, with the molecular transition dipole orientated almost perpendicular to the nanocavity field. Therefore, the coupling strength is relatively weaker in this case compared to the situation with the molecule in the center of the nanocavity and oriented along the local near fields, thus leading to a reduced value of the effective Purcell factor.

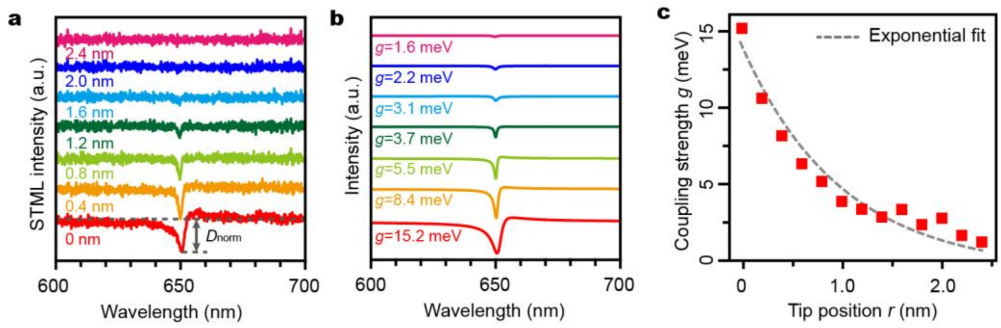

Supplementary Figure 4 | Normalized Fano spectra as a function of lateral tip position. a, Normalized experimental STML spectra (−2.5 V, 200 pA, 20 s) at different tip positions as indicated in the labels of each spectrum. The distance is referred to the position reference r=0. The value of the normalized dip depth can be obtained directly from the spectra, as indicated by an arrow between the dashed lines as an example for position r=0. The spectral normalization process is carried out

through dividing the Fano spectra (as shown in main-text Fig. 2b) by the plasmonic background acquired on the NaCl/Ag surface. **b**, Fitted normalized Fano spectra with the coupling strength *g* labelled for each spectrum corresponding to **a**. **c**, Variations of the coupling strength *g* as a function of tip position *r*. The exponential fit to the experimental values is also shown with a dashed line, showing a decay length of 0.96 ± 0.07 nm.

Supplementary Note 5. Tip-height dependent fluorescence and Fano spectra

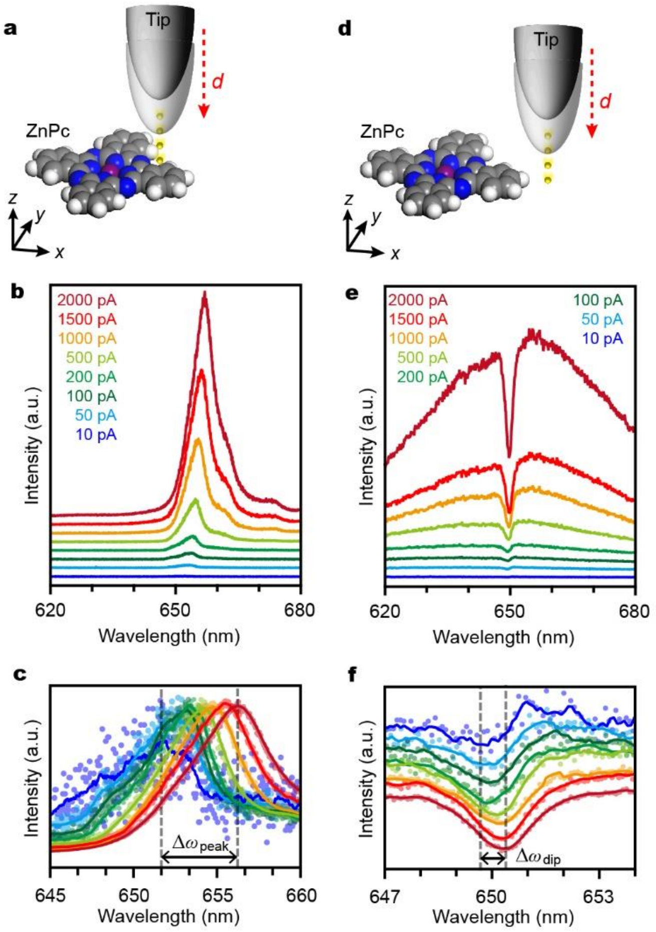

Supplementary Figure 5 | Tip-height dependent fluorescence and Fano spectra of a single ZnPc molecule. a, Schematic of the molecule-tip system for the generation of molecular fluorescence by positioning the tip on top of the molecule. As the tunneling

current increases, the tip is moved closer to the molecule. **b**, Exponential fluorescence spectra of the ZnPc molecule for tunneling currents varying from 10 pA to 2000 pA. **c**, Selected spectral range of the spectra in **b** with the intensities normalized to their maximum, highlighting a peak shift ($\Delta\omega_{peak}$) of about 4.6 nm. **d**, Schematic of the molecule-tip system for the generation of Fano resonance by positioning the tip in close proximity to the molecule and making vertical displacement. **e**, Fano spectra for the tip positioning in **d**, with the same tunnelling currents as in **b**. **f**, Selected spectral range of the Fano spectra in **e** with the intensities normalized to its plasmonic background. A photonic Lamb shift ($\Delta\omega_{dip}$) of ~0.6 nm (~1.7 meV) can be observed (dashed lines).

When the tip is positioned on top of the molecule (Supplementary Figs. 5a–5c**,** similar to situation I in main-text Fig. 1), molecular fluorescence is generated by the excitation of the molecule directly from tunneling electrons. In this situation, the red shift observed for the emission peak along with the increase of tunneling currents (and thus the decrease of the tip–molecule distance) is also an effect of the photonic Lamb shift due to increased molecule–plasmon interaction. Consistent with an increased interaction with the plasmonic nanocavity, the shift is also found to be larger than when the tip is located at the edge of the molecule (similar to situation II in main-text Fig. 1). This is illustrated in Supplementary Figs. 1c and 1d by the further red-shift of the emission peak position compared to the Fano dip position.

The consistency of the Lamb shift can be further tested by varying the vertical distance between the tip and substrate at the edge of molecule (see Supplementary Fig. 5d). As observed in Supplementary Figs. 5e and 5f, the Fano dip position is shifted

further to red when the tunneling current is increased (i.e., the tip height is decreased) and the local plasmonic field is strengthened. This is again consistent with the evolution of the photonic Lamb shift as the molecule-plasmon interaction is increased when the tip is moved laterally towards the molecule (main-text Fig. 2c).

Supplementary Note 6. Detuning analysis of Fano spectra

As discussed in Supplementary Note 2 and Note 3, the symmetric features of the Fano lineshape can be described by the Fano parameter q. This parameter takes a particularly simple form in the case of our dipole model in Supplementary Note 2:

$$q = -\frac{\alpha_{\mathrm{m}}^{0}\omega_{\mathrm{m}}^{2}\,\mathrm{Re}\left[G_{\mathrm{mp}}\alpha_{\mathrm{p}}G_{\mathrm{pm}}\right]}{\omega_{\mathrm{m}}\gamma_{\mathrm{m}}^{0}+\alpha_{\mathrm{m}}^{0}\omega_{\mathrm{m}}^{2}\,\mathrm{Im}\left[G_{\mathrm{mp}}\alpha_{\mathrm{p}}G_{\mathrm{pm}}\right]} \approx -\frac{\alpha_{\mathrm{m}}^{0}\omega_{\mathrm{m}}\,\mathrm{Re}\left[G_{\mathrm{mp}}\alpha_{\mathrm{p}}G_{\mathrm{pm}}\right]}{\gamma_{\mathrm{m}}}. \tag{34}$$

Here we have used the definition of γ_{m} in Eq. (21). Inserting the expression for the polarizability of the plasmonic dipole in Eq. (9) into the above equation, under first-order approximation we can obtain

$$q \approx -\frac{2}{\gamma_{\mathrm{m}}}\left(\frac{\omega_{\mathrm{p}}}{\gamma_{\mathrm{p}}}\right)^{2}\alpha_{\mathrm{m}}^{0}\alpha_{\mathrm{p}}^{0}G_{\mathrm{mp}}G_{\mathrm{pm}}\Delta\,, \tag{35}$$

where $G_{\mathrm{mp}}G_{\mathrm{pm}}$ is real when using the expression of the near-field Green's function for a dipole. Thus within this dipole model, the Fano parameter is proportional to the detuning Δ, and q would thus be zero when the plasmon and the molecule are in resonance, i.e., q=0 for zero detuning condition (Δ=0).

For the generalized model that includes higher order interactions and spatial distribution of the molecular transition dipole, it is difficult to get the explicit expression of q as a function of detuning because of the complexity of the plasmonic response.

Under the simplest assumption, the field enhancement term $G_{\mathrm{pm}}\overleftrightarrow{\mathbf{M}}$ can be described by a modified Lorentzian lineshape in terms of the plasmonic resonant frequency ω_{p} and the decay rate γ_{p} as:

$$G_{\mathrm{pm}}\overleftrightarrow{\mathbf{M}} = G_{\mathrm{pm}}^{0} M_0 \frac{\omega_{\mathrm{p}}^2}{\omega_{\mathrm{p}}^2 - \omega^2 - i\omega\gamma_{\mathrm{p}}}, \qquad (36)$$

where M_0 can be a complex number with generality when higher-order interactions are taken into account. By combing Eqs, (36), (18) and (20) with Eq. (26), under first-order approximation we can obtain

$$q \approx -\frac{2}{\gamma_{\mathrm{m}}}\left(\frac{\omega_{\mathrm{p}}}{\gamma_{\mathrm{p}}}\right)^2 \alpha_{\mathrm{m}}^{0}\,\mathrm{Re}\left[G_{\mathrm{pm}}^{0} M_0\left(\Delta + i\frac{\gamma_{\mathrm{p}}}{2}\right)\right]. \qquad (37)$$

This expression shows a similar linear relation between q and Δ as in Eq. (35), however, the value of $G_{\mathrm{pm}}M_0$ would be now a complex number with generality rather than a real number as in the dipole model. Therefore, an additional contribution to the Fano parameter emerges, resulting in a non-zero value of q even for the zero-detuning condition.

Supplementary Fig. 6a shows the experimental spectra normalized to its background for different detuning conditions at a given tip position reference r=0 (the data in Supplementary Fig. 6a are the same as in main-text Fig. 3a). Supplementary Fig. 6b show the corresponding fitted Fano lineshape obtained from Eq. (25), with the value of the Fano parameter q indicated. Supplementary Figs. 6c and 6d show similar processing of the experimental and fitted Fano spectra for the tip position r=0.4 nm. Both experimental and theoretical results in Supplementary Fig. 6 show clearly a non-zero q value at the zero-detuning condition (Δ=0), suggesting the occurrence of an asymmetric

Fano dip. In addition, the Fano spectra do not exhibit "mirror-symmetry" for the same amount of positive and negative detunings. In the present system, the complex nature of the term $G_{pm}M_0$ in Eq. (37) and the resultant asymmetric Fano dip at zero-detuning is a fingerprint of the complexity of the interaction that can be related to the actual spatial distributions of the molecular transition dipole, higher-order plasmonic modes,[11] or any other mechanisms beyond the dipole model of interaction introduced in Supplementary Note 2. We note that in other systems with direct external excitation of the molecules, an asymmetry could be produced within a dipole-dipole interaction model, however this is not the case in our conditions where the molecule is far enough so that it is not directly excited by the electron beam.

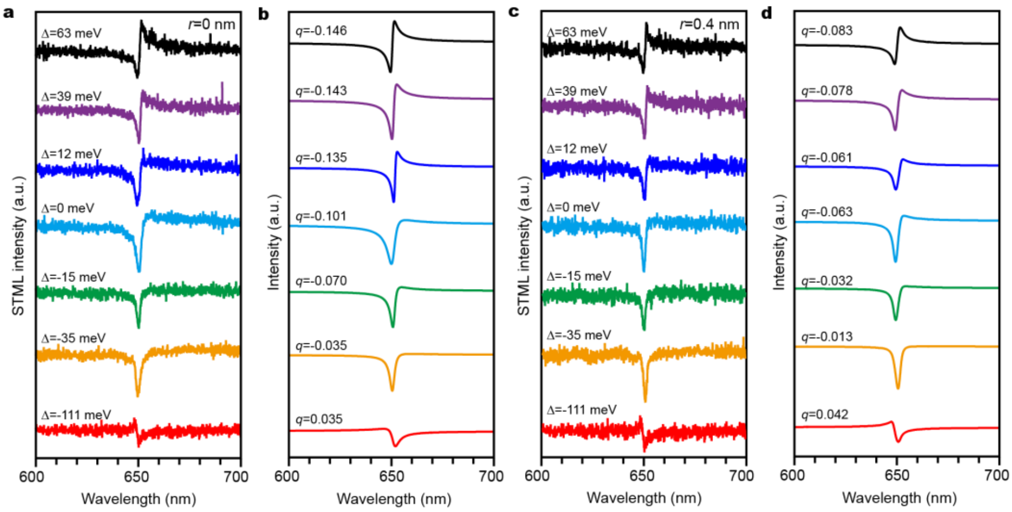

Supplementary Figure 6 | Normalized Fano spectra for different detunings. a, Normalized experimental STML spectra (−2.5 V, 200 pA, 20 s) for tip position r=0 at different detunings, showing the transition of symmetries of the Fano lineshape. **b**, Fitted normalized Fano spectra corresponding to those in **a**, with the Fano parameters q labelled in each spectrum. **c**, Normalized experimental STML spectra (−2.5 V, 200 pA, 20 s) for the tip position r=0.4 nm and **d**, the corresponding fitted Fano spectra.

An important aspect of the interaction between a single molecule and nanocavity plasmon is the estimation of the "splitting" of the peak-maximum in the Fano spectra for different detuning conditions, as depicted in main-text Fig. 3d. If the system is in the strong coupling regime, the splitting of the peaks corresponds to the eigen-modes of the coupled system. If the dampings of molecule and plasmon are ignored, the eigen-frequencies ($\omega_{U,L}$) for an upper and lower branch can be expressed as:[12]

$$\omega_{\mathrm{U,L}} = \frac{1}{2}\left(\omega_{\mathrm{m}} + \omega_{\mathrm{p}}\right) \pm \frac{1}{2}\sqrt{\Delta^2 + 4g^2}\,. \tag{38}$$

The so-called Rabi splitting is usually defined as $\Omega_{\mathrm{s}} = \omega_{\mathrm{U}} - \omega_{\mathrm{L}} = 2g$ at zero detuning. Although the interaction between the nanocavity plasmon and the molecule in the present system is not strong enough to result in a peak splitting with two hybridized states, the two peak-maximum positions in the emission spectra can be used to estimate the magnitude of this coupling strength. By using Eq. (38) to fit the experimental data of the peak-maxima in the Fano spectra in main-text Fig. 3d, we can obtain the two solid lines shown in main-text Fig. 3d, with an energy "splitting" Ω_s as large as 32.6 meV.

Supplementary Note 7. Orientation dependence of Fano dip position

Owing to the vectorial nature of both the molecular transition dipole moment and the local plasmonic field, the coherent coupling between the molecule and the nanocavity plasmon could also be affected if the nanocavity is placed in different orientations with

respect to the single molecule, resulting in a shift of the Fano dip position (i.e. the Lamb shift). Since the ZnPc molecule has D_{4h} symmetry, there exist two equivalent orthogonal transition dipole moments, i.e., $\mathbf{p}_{\mathrm{m}}^{x}$ along one lobe direction in x-axis and $\mathbf{p}_{\mathrm{m}}^{y}$ along the other lobe direction in y-axis, as schematically shown in main-text Fig. 4a. Therefore, it is necessary to consider both $\mathbf{p}_{\mathrm{m}}^{x}$ and $\mathbf{p}_{\mathrm{m}}^{y}$ molecular dipole moments in the model. The coupling equations of Eq. (15) can be written in this situation as:

$$\begin{cases} \mathbf{p}_{\mathrm{m}}^{x} = \alpha_{\mathrm{m}}^{x}\left(\overleftrightarrow{\mathbf{M}}^{x}\cdot\mathbf{E}_{\mathrm{j}} + \overleftrightarrow{\mathbf{G}}_{\mathrm{mm}}^{xx}\cdot\mathbf{p}_{\mathrm{m}}^{x} + \overleftrightarrow{\mathbf{G}}_{\mathrm{mm}}^{xy}\cdot\mathbf{p}_{\mathrm{m}}^{y}\right) \\ \mathbf{p}_{\mathrm{m}}^{y} = \alpha_{\mathrm{m}}^{y}\left(\overleftrightarrow{\mathbf{M}}^{y}\cdot\mathbf{E}_{\mathrm{j}} + \overleftrightarrow{\mathbf{G}}_{\mathrm{mm}}^{yy}\cdot\mathbf{p}_{\mathrm{m}}^{y} + \overleftrightarrow{\mathbf{G}}_{\mathrm{mm}}^{yx}\cdot\mathbf{p}_{\mathrm{m}}^{x}\right) \\ \mathbf{p}_{\mathrm{p}} = \alpha_{\mathrm{p}}\left(\mathbf{E}_{\mathrm{j}} + \overleftrightarrow{\mathbf{G}}_{\mathrm{pm}}^{x}\cdot\mathbf{p}_{\mathrm{m}}^{x} + \overleftrightarrow{\mathbf{G}}_{\mathrm{pm}}^{y}\cdot\mathbf{p}_{\mathrm{m}}^{y}\right) \end{cases}. \tag{39}$$

We can obtain the solution for the plasmon dipole $\mathbf{p}_{\mathrm{p}}$ as

$$\mathbf{p}_{\mathrm{p}} = \alpha_{\mathrm{p}}\left(\overleftrightarrow{\mathbf{I}} + \frac{\overleftrightarrow{\mathbf{G}}_{\mathrm{pm}}^{x}\cdot\left(\overleftrightarrow{\mathbf{M}}^{x} + \dfrac{\overleftrightarrow{\mathbf{G}}_{\mathrm{mm}}^{xy}\mathbf{M}^{y}}{\left(\alpha_{\mathrm{m}}^{y}\right)^{-1} - \overleftrightarrow{\mathbf{G}}_{\mathrm{mm}}^{yy}}\right)}{\left(\alpha_{\mathrm{m}}^{x}\right)^{-1} - \overleftrightarrow{\mathbf{G}}_{\mathrm{mm}}^{xx} - \dfrac{\overleftrightarrow{\mathbf{G}}_{\mathrm{mm}}^{xy}\overleftrightarrow{\mathbf{G}}_{\mathrm{mm}}^{yx}}{\left(\alpha_{\mathrm{m}}^{y}\right)^{-1} - \overleftrightarrow{\mathbf{G}}_{\mathrm{mm}}^{yy}}} + \frac{\overleftrightarrow{\mathbf{G}}_{\mathrm{pm}}^{y}\cdot\left(\overleftrightarrow{\mathbf{M}}^{y} + \dfrac{\overleftrightarrow{\mathbf{G}}_{\mathrm{mm}}^{yx}\mathbf{M}^{x}}{\left(\alpha_{\mathrm{m}}^{x}\right)^{-1} - \overleftrightarrow{\mathbf{G}}_{\mathrm{mm}}^{xx}}\right)}{\left(\alpha_{\mathrm{m}}^{y}\right)^{-1} - \overleftrightarrow{\mathbf{G}}_{\mathrm{mm}}^{yy} - \dfrac{\overleftrightarrow{\mathbf{G}}_{\mathrm{mm}}^{yx}\overleftrightarrow{\mathbf{G}}_{\mathrm{mm}}^{xy}}{\left(\alpha_{\mathrm{m}}^{x}\right)^{-1} - \overleftrightarrow{\mathbf{G}}_{\mathrm{mm}}^{xx}}}\right)\mathbf{E}_{\mathrm{j}}. \tag{40}$$

Considering the vectorial nature of the molecular transition dipole and the local plasmonic field, the values of $\overleftrightarrow{\mathbf{G}}_{\mathrm{mm}}^{xy(yx)}, \overleftrightarrow{\mathbf{G}}_{\mathrm{mm}}^{xx(yy)}$, $\overleftrightarrow{\mathbf{G}}_{\mathrm{pm}}^{x(y)}$ and $\overleftrightarrow{\mathbf{M}}^{x(y)}$ would be all angular dependent, but cannot be explicitly expressed. In order to obtain a simple estimation of the angular dependence of these interaction terms, we treat the tip-substrate plasmonic dipole as a point dipole and estimate its interaction with the transition dipoles of the molecule in both directions ($\mathbf{p}_{\mathrm{m}}^{x}$ and $\mathbf{p}_{\mathrm{m}}^{y}$). The spatial distributions of the two equivalent transition dipoles of the ZnPc molecule are calculated from the density functional theory,[1,13] as shown in Supplementary Fig. 7a. In Supplementary Fig. 7b, we plot the normalized values of the interaction energy between the point dipole and the

molecular transition dipoles as a function of the orientation angle (θ) for a set of different given lateral separations (relative to the molecular center). When the point dipole is far away from the molecule (black line for a distance of 3.0 nm), the interaction is independent of the orientation (θ) of the point dipole, thus losing the information of the internal structure of the molecule. However, as the point dipole gets closer to the molecule, the interaction energy is found to be strongly dependent on the point-dipole orientation angle (red line for the shorter separation distance of 1.0 nm). The spatial distribution of the interaction energy reveals the structural symmetry of the molecule, specifically a four-fold symmetry associated with the D_{4h} point group for the ZnPc molecule. In this sense, for a molecule with lower symmetry, different Fano dip positions are expected to be observed in the angular-dependent Fano spectra due to the non-equivalent nature of specific transition dipole moments of a single molecule along different directions. Therefore, the single-molecule Fano spectra can be used to reveal the orientation of transition dipole moments for a single molecule and associated transition energies as well as the character of frontier orbitals involved in the transitions.

In order to reproduce the symmetry pattern illustrated in Supplementary Fig. 7b, we consider different angular dependences of the interaction terms described above. For the mutual-interaction term $\overleftrightarrow{\mathbf{G}}_{\mathrm{pm}}^{x(y)}$ and enhancement factor $\overleftrightarrow{\mathbf{M}}^{x(y)}$ we can assume a $\cos\theta$ ($\sin\theta$) dependence, following the typical distribution of the field for a fixed dipole. For the cross-interaction terms $\overleftrightarrow{\mathbf{G}}_{\mathrm{mm}}^{xy(yx)}$ and the self-interaction terms $\overleftrightarrow{\mathbf{G}}_{\mathrm{mm}}^{xx(yy)}$ higher-order dependence $\cos^n\theta\sin^n\theta$ and $\cos^{2n}\theta$ ($\sin^{2n}\theta$) relations are assumed, respectively. Supplementary Fig. 7c shows simulated Fano spectra when considering different n

values in Eqs. (10) and (40). For n=1 (similar to considering the equivalent molecular dipoles as point dipoles), the Fano spectra show negligible angular dependence. If we consider a larger value of n (similar to considering higher-order terms in the response of the equivalent molecular dipoles), the shift of the Fano dip position reveals a four-fold symmetry on the orientation angle θ, consistent with the symmetry pattern shown in Supplementary Fig. 7b for a shorter separation distance of the probing tip dipole (green and red lines). We find that considering n=2 provides good agreement with the experimentally observed evolution of the Fano dip (main-text Fig. 4c).

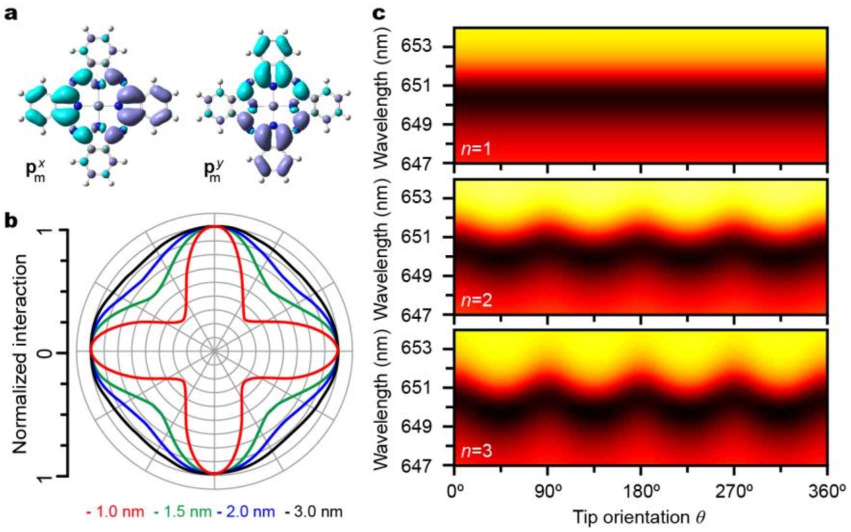

Supplementary Figure 7 | a, Spatial distributions of the transition dipoles $\mathbf{p}_{\mathrm{m}}^{x}$ and $\mathbf{p}_{\mathrm{m}}^{y}$ for a ZnPc molecule. Positive and negative charge densities are represented by the violet and cyan shading, respectively, which are superimposed on the ball-and-stick model of a ZnPc molecule (the H, C, N and Zn atoms are represented by the white, grey, blue and violet spheres, respectively). **b**, Polar plot of the interaction between a point dipole and the transition dipoles shown in **a**. The interaction strength has been normalized to its maximum values for different distances relative to the molecular center: 1.0 nm (red), 1.5 nm (green), 2.0 nm (blue) and 3.0 nm (black), respectively. **c**,

Simulated color plots of the Fano spectra considering different orders of self- and cross-interaction terms (n=1, 2 and 3, respectively). The parameters used for the spectrum calculation are obtained from the fit to the experimental spectrum for θ=0 at zero detuning (the same spectrum as that shown in main-text Fig. 4b).

The Fano spectra as a function of tip orientation (θ) for different detuning conditions are also simulated for n=2, as shown in Supplementary Fig. 8. A remarkable agreement between the simulated and experimental spectra can be obtained. A periodic pattern with four periods corresponding to the four-lobe feature of the molecule can be always observed for either positive, zero or negative detuning, which indicates that the Fano dip, as well as the related Lamb shift, is sensitive to the relative orientations between the molecular dipole and the local plasmonic field.

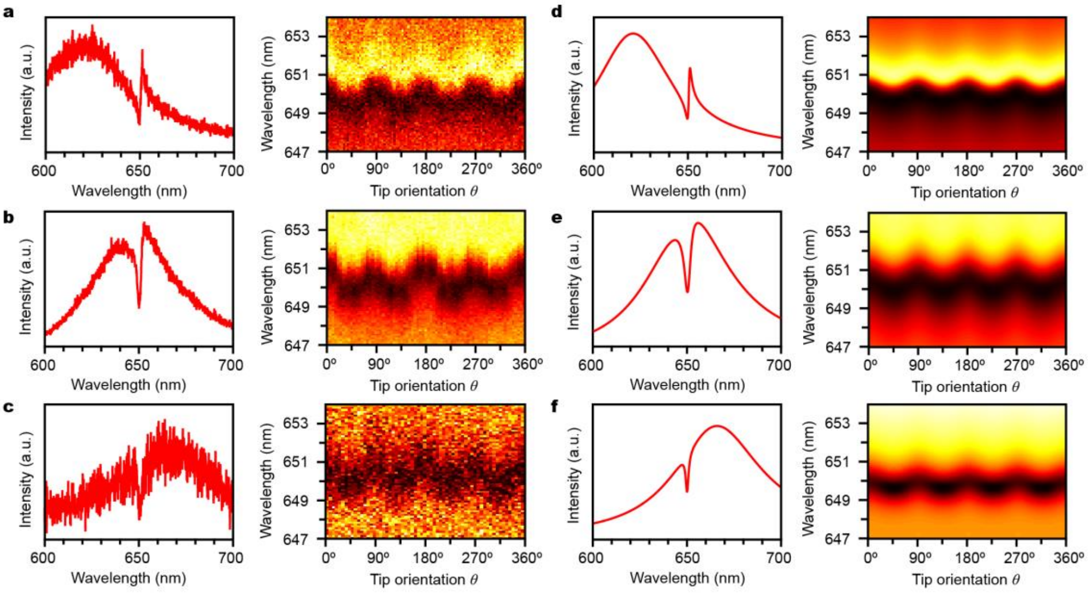

Supplementary Figure 8 | Dependence of Fano dip position as a function of tip orientations (θ) for different detunings. a−c, Typical experimental Fano spectra (left) and corresponding color plots of STML spectral intensity around the Fano dip

(right) (−2.5 V, 200 pA, 5 s) showing periodic variations of the Fano dip from θ=0 to 360º at different detunings (top: Δ>0, middle: Δ=0, bottom: Δ<0). **d−f**, Theoretical simulations of the Fano spectra corresponding to **a−c** by using Eqs. (10) and (40).

Supplementary References

1. Zhang, Y. *et al.* Visualizing coherent intermolecular dipole–dipole coupling in real space. *Nature* **531**, 623-627 (2016).
2. Hone, D., Mühlschlegel, B. & Scalapino, D. Theory of light emission from small particle tunnel junctions. *Appl. Phys. Lett.* **33**, 203-204 (1978).
3. Bigourdan, F., Hugonin, J.-P., Marquier, F., Sauvan, C. & Greffet, J.-J. Nanoantenna for Electrical Generation of Surface Plasmon Polaritons. *Phys. Rev. Lett.* **116**, 106803 (2016).
4. Novotny, L. & Hecht, B. *Principles of nano-optics*. (Cambridge university press, 2012).
5. Aizpurua, J., Apell, S. P. & Berndt, R. Role of tip shape in light emission from the scanning tunneling microscope. *Phys. Rev. B* **62**, 2065-2073 (2000).
6. Zhou, X., Ren, A. M., Feng, J. K. & Liu, X. J. Theoretical studies on the one-and two-photon absorption of tetrabenzoporphyrins and phthalocyanines. *Can. J. Chem.* **82**, 19-26 (2004).
7. Shah, R. A., Scherer, N. F., Pelton, M. & Gray, S. K. Ultrafast reversal of a Fano resonance in a plasmon-exciton system. *Phys. Rev. B* **88**, 075411 (2013).
8. Esteban, R., Aizpurua, J. & Bryant, G. W. Strong coupling of single emitters interacting with phononic infrared antennae. *New J. Phys.* **16**, 013052 (2014).
9. Yao, P. *et al.* Ultrahigh Purcell factors and Lamb shifts in slow-light metamaterial waveguides. *Phys. Rev. B* **80**, 195106 (2009).
10. Auffèves, A. *et al.* Controlling the dynamics of a coupled atom-cavity system by pure dephasing. *Phys. Rev. B* **81**, 245419 (2010).
11. Joe, Y. S., Satanin, A. M. & Kim, C. S. Classical analogy of Fano resonances. *Phys. Scr.* **74**, 259-266 (2006).
12. Rudin, S. & Reinecke, T. Oscillator model for vacuum Rabi splitting in microcavities. *Phys. Rev. B* **59**, 10227 (1999).
13. Gaussian 09 (Gaussian, Inc., Wallingford, CT, USA, 2009).
